# Social group algorithm-based MPPT coupled with phase shift resonant converter for battery charging through partially shaded PV systems

**DOI:** 10.1038/s41598-025-31674-y

**Published:** 2026-03-18

**Authors:** Jayachitra Jayaraman, Sridhar Ramasamy, Srinivasan Vadivel, S. Thangavel, Hassan Abdurrahman Shuaibu, Taha Selim Ustun

**Affiliations:** 1 Department of Electrical and Electronics Engineering, SRM Institute of Science and Technology, Kattankulathur, Tamil Nadu 603 203 India; 2 Department of Electronics and Instrumentation Engineering, SRM Valliammai Engineering College, Kattankulathur, Tamil Nadu 603 203 India; 3 Department of Electrical and Electronics Engineering, National Institute of Technology, Puducherry, 609 609 India; 4 Department of Electrical, Telecommunications and Computer Engineering, Kampala international university, Kampala, Uganda; 5 AIST (FREA), Fukushima Renewable Energy Institute, National Institute of Advanced Industrial Science and Technology (AIST), Fukushima, Koriyama 9630298 Japan

**Keywords:** Maximum power point tracking, Partially shading, Phase shifted modulation, Battery charging, Social group optimisation (SGO), Resonant converter, Energy storage, Renewable energy, Electrical and electronic engineering

## Abstract

The paradigm shift toward electric transportation is a necessary step in mitigating greenhouse gas discharges in connection with the internal combustion engine emissions. Nevertheless, the Electric Vehicle (EV) charging infrastructures predominantly rely on fossil fuel-based power generation, which again aggravates climate change. Imparting renewable energy sources for powering the charging systems is therefore essential, with photovoltaic (PV) power standing out as a scalable and portable solution. In PV-based charging setups, the power available from the panels can vary widely, especially when some modules fall under shade. To keep the charging process steady during such conditions, a smarter MPPT approach becomes necessary. In this work, a single-stage full-bridge converter operating with phase-shift control is combined with an Social group optimization based MPPT method to improve how the system reacts to these fluctuations. The converter has been designed so that the switches achieve soft-switching, which helps in cutting down losses and keeping the output voltage steady over different operating points. A 3 kW prototype was built and tested along with detailed simulations. Both sets of results show that the converter, together with the MPPT strategy, is able to draw consistent power from the PV array and continue charging the battery smoothly even when the sunlight changes abruptly. The system achieves a peak efficiency of 97%, representing a notable improvement over conventional dual-stage system. Additionally, the output voltage regulation is enhanced by 2%, demonstrating the viability of the proposed converter-MPPT architecture for future PV-powered EV charging stations with improved energy conversion efficiency and resilience under environmental uncertainties.

## Introduction

Electric Vehicle fleet is fast emerging across the globe. In Indian sub-continent perspective, the growth of EV has its impact on the promises rendered in COP 26^1^. Attaining net zero commitment by 2070 is ambitious, but with the given development in the EV infrastructure and rigorous policies for renewable source deployment, it is not a distant dream to attain net zero^2^. Among the renewable sources, the photovoltaic (PV) and Fuel cell (FC) are very compatible with EV drive and charging infrastructure. In fact, FC will be a very good candidate for direct EV drives as a coveted source^3^ as the energy density factor is very high. However, the high costs and complex technologies involved in hydrogen storage and handling make it a less pragmatic candidate for many applications. PV, on the other hand will be a handy source to be deployed in charging stations for two wheelers and three wheelers so that the grid power reliance can be reduced considerably^4^. The lack of charging infrastructure in developing economies poses stiff challenges for EV proliferation. The cost involved in building charging infrastructure as well as relevant communication protocols play a crucial role^5,6^. Moreover, if the charging infrastructures are levied only from grid power, the mission of net-zero will face a major setback, as the predominant share of power is generated from fossil fuels such as coal. Therefore, the charging infrastructures can be built with PV array as source which makes the EV sector greener. But the major issues here are the inherent intermittency that PV possesses in nature. The output power-voltage (P–V) characteristics of a PV array are nonlinear and exhibit a unique maximum power point (MPP) corresponding to the optimal combination of voltage and current. MPPT algorithms are employed to identify this point in real time, using power electronic converters to regulate the operating point of the PV system^7^. While the impact of temperature on power output is relatively modest due to its logarithmic influence, irradiation plays a dominant role as it has a near-linear relationship with the output power. In most PV systems, the MPPT controller adjusts the duty cycle of the converter to locate the point at which the array delivers its highest power. The commonly used P&O and INC methods work reasonably well when the sunlight is uniform across the panel surface^8,9^. P&O changes the operating voltage step by step and checks whether the power moves up or down, but it tends to keep oscillating around the best operating point. INC improves this behaviour by using the slope of the P–V curve to decide the direction of movement, although the method requires more computation because it relies on derivative information.

When part of the array is shaded, the output power curves develop several small peaks, and this makes the tracking process far more complicated. Under these conditions, both the basic and enhanced versions of P&O and INC often end up locking onto one of the local peaks instead of the true global maximum. A variety of MPPT schemes have been reported in literature, but many of them still struggle when the irradiance changes rapidly or when severe shading occurs. Issues such as slow response, unnecessary oscillations, and failure to move out of local traps remain common, which underlines the need for MPPT techniques that are more flexible and capable of handling such irregular operating conditions^10^. To overcome the limitations of conventional MPPT methods under partial shading, research has increasingly focused on global search, bio-inspired, and Artificial Intelligence (AI) based algorithms^11^. These intelligent techniques are broadly classified into evolutionary (e.g., Genetic Algorithm, Differential Evolution) and bionic approaches, with the former gaining prominence in the 1990s for their population-based search and adaptability. Evolutionary algorithms like Genetic Algorithm (GA) and Differential Evolution (DE) rely on initialization, crossover, and mutation based on the principle of survival of the fittest^12,13^. The Particle Swarm Optimization (PSO), inspired by swarming behaviour of birds and fish, remains to be the most effective among the global search algorithms^14^. The reason for its relevance till date is its optimum convergence accuracy and simple implementation. But, when the irradiation pattern tends to vary rapidly, the algorithm at times stagnates at a pseudo- peak. Therefore, the exploration on proposing inventive algorithms is at steady pace. The Grey Wolf Optimization (GWO)^15^, inspired by the hunting adoption of leadership hierarchy and hunting strategy of grey wolves, tries to balance both exploration and exploitation. This facilitates rational evading of local maxima during the search process. But here too, large steady-state oscillations prevail under dynamic insolation pattern. Another interesting algorithm, Hippopotamus Algorithm (HOA)^16^ is emulated by natural behaviour of hippopotamus. The tracking efficiency is high, but it involves higher computational effort. Other algorithms like MFO (Moth Flame Optimization) and Cuckoo Search Algorithm (CSA) have also been tried and tested, but these algorithms exhibit poor tracking reliability under fast-changing irradiance^17^. The recent advancement in deep learning computation has also been deployed in MPPT through DLCI (Deep Learning and Cognitive Inspired) neural decision models and learning architectures. The advantage is the speed of tracking is swift under learned conditions, but on the other hand, requires large training data. Artificial Bee Colony (ABC), and Grey Wolf Optimization (GWO) offer improved global search capabilities^18^, but face issues like slow convergence and increased complexity. Enhanced variants like E-PSO have attempted to address these limitations using fast-response digital signal processing (DSP) controllers^19^. Ant Colony Optimization (ACO), inspired by the foraging behaviour of ants, is valued for its ability to explore complex solution spaces and avoid local maxima due to its collective intelligence mechanism^20^. However, its sluggish response in highly dynamic irradiance conditions limits its suitability for real-time MPPT applications where fast convergence is critical. Despite the individual strengths observed in various global MPPT strategies, the recurring limitations such as slow convergence, local trapping, or high computational burden warrant the exploration of more adaptive solutions. In this context, Social Group Optimization (SGO) has been employed in the present work due to its proven capability to balance exploration and exploitation through socially driven interactions^21^. Social Group Optimization (SGO) is chosen as the MPPT strategy because its two-phase search mechanism (improving and acquiring phases) provides a strong balance between exploration and exploitation, which is essential for reliably locating the global maximum power point (GMPP) in multi-peaked P–V curves under partial shading. The algorithm is parameter-lean, requiring only a self-introspection factor, which reduces implementation complexity compared to other global search techniques. Its update rules are computationally efficient and easily mapped to the PSFB control framework, making it suitable for embedded real-time applications. Benchmark studies demonstrate that SGO achieves competitive or superior solutions with fewer fitness evaluations than many existing metaheuristic counterparts, which directly benefits MPPT tasks where iteration budgets are constrained. Moreover, the mechanism inherently mitigates steady-state oscillations by guiding particles based on both the global best and peer influence, yielding faster convergence with stable operation.

Resonant converters are an appropriate choice for battery charging, as zero-voltage switching (ZVS) and zero-current switching (ZCS) are achieved^22^. The non-isolated topologies of resonant converters are least preferred due the concerns like electromagnetic interference, increased common mode noise, and need for competent protection due to the absence of galvanic isolation^23^. Among the isolated topologies, the full bridge converter is advantageous, as it can handle high-power by utilizing the entire transformer during operation. Besides that, the four switches in the topology reduces the current stress on individual components, leading to lower conduction losses^24^. Apart from full bridge there are numerous topologies of resonant converters, but the prudent choice needs to be based on the power capacity, efficiency requirement and specific application. The half bridge circuit possess lesser number of switches, but the power handling capacity is less. The typical buck, boost converters also quite suitable for the charging circuits but the lack of galvanic isolation raises protection and common mode noise issues. The flyback topology provides galvanic isolation, but the single-switch design adds stress, and the full potential of the ferrite-core transformer cannot be utilized due to inevitable two-phase charging and discharging operations. The interleaved topology is better but the increased in components and control complexity will exist. The push-pull topology is apt for high power as transformer is centrally tapped and two switches are employed. The transformer core saturation will happen when the controller is not prudently chosen, the full bridge converter provides a robust solution for battery charging due to its efficiency, flexibility, and isolation capabilities.

**Fig. 1:**
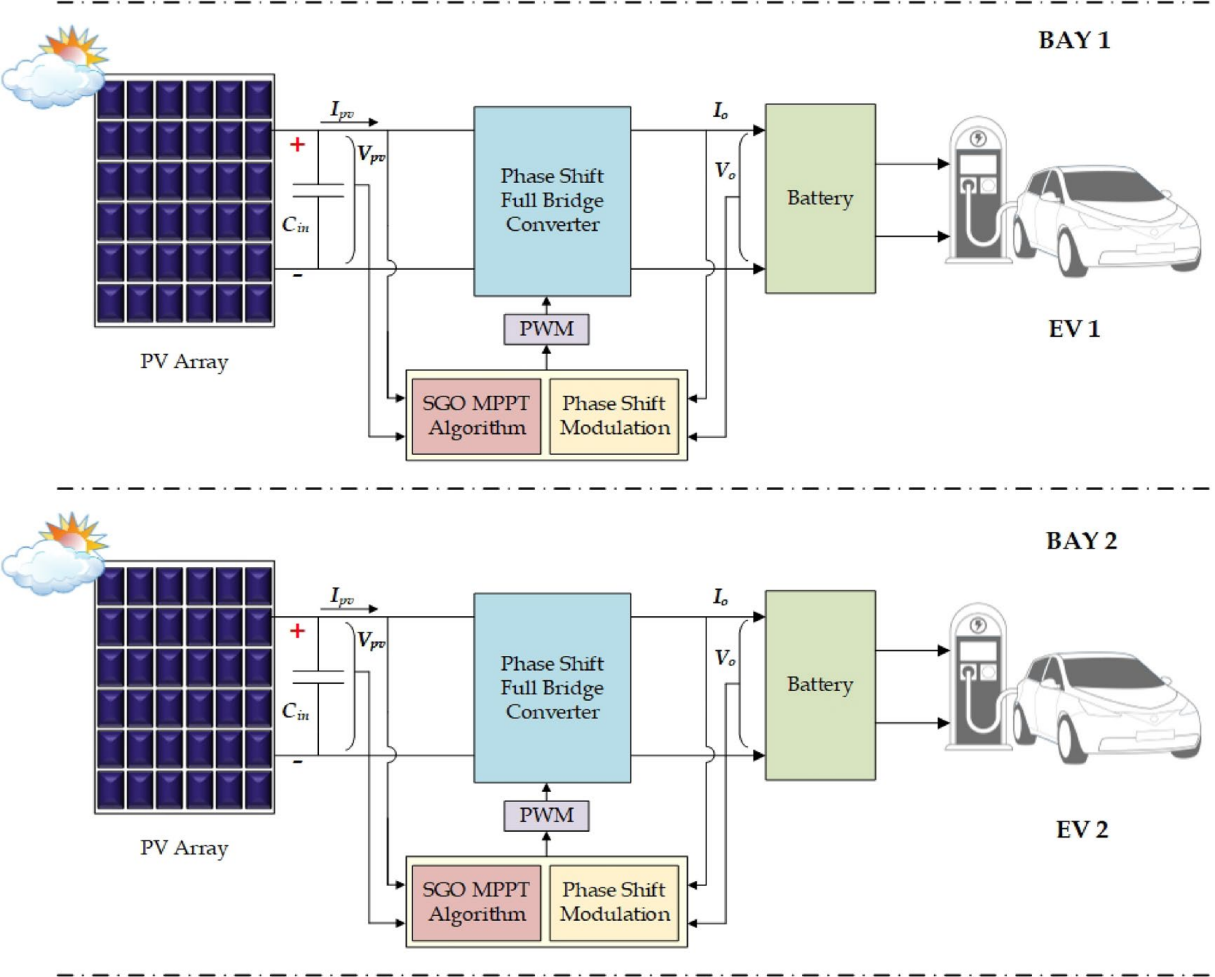
PV aided EV charging station.

The selection of an appropriate control strategy is equally crucial for optimizing the performance and efficiency. The advanced controllers like sliding mode control and model predictive control can handle multi variable problems and could excel in non-linear changes in the system, but when it comes to implementation, high expertise is in demand for handling the coding complexity. However, these controllers are reliable for real-time forecasting and dynamic optimization, and they establish good response under rapidly changing environmental and load conditions^25^. Fuzzy logic control (FLC) provides flexibility and handles uncertainty well but the execution and the output reliability depend on the versatility of the rule set. Adaptive control scheme is very adjustable to dynamic changes in the system, but the design complexity is high. Among all control schemes the phase shift modulation stands out as a particularly effective control strategy for full-bridge converters used in battery charging^26^. To regulate the output voltage and efficient power transfer the phase difference between the two halves of the converter have been adjusted. It provides superior efficiency, reduced component stress, and excellent performance across a wide range of operating conditions, making it a highly advantageous choice in this context. Figure 1 presents a typical charging infrastructure with PV, resonant power electronic converter, and advanced MPPT controller cuddled with modulation schemes. This hybrid control scheme facilitates competitive maximum power tracking during shading as well ensures optimized battery charging. While numerous MPPT techniques and power converter topologies have been proposed independently, an integrated strategy that robustly handles both global tracking under partial shading and high-efficiency power conversion remains underexplored. Although a wide range of MPPT algorithms (P&O, INC, PSO, GWO, HOA, MFO, CSA, ACO, ABC, DE, DL-based methods, etc.) and several DC-DC converter topologies such as buck, buck-boost, flyback, interleaved, half bridge and full-bridge have been extensively studied, these two domains are largely explored independently. Existing MPPT-focused works primarily address global peak tracking under partial shading but do not consider how the chosen converter influences switching behaviour, soft-switching windows, or power-transfer efficiency. Conversely, converter-oriented studies optimise ZVS/ZCS operation and efficiency but overlook the effect of dynamic and multi-peaked PV characteristics on control stability and energy extraction. The research gap lies in the lack of a unified and coordinated co-design approach that simultaneously integrates a global MPPT technique with a high-efficiency resonant converter for EV battery charging, especially under rapidly varying irradiance and partial shading. This missing coordination results in sub-optimal system performance when both global tracking accuracy and converter soft-switching requirements must be satisfied concurrently. Although numerous MPPT strategies and converter control methods have been explored individually, there are no studies that bring a socially inspired global MPPT algorithm and a phase-shift full-bridge (PSFB) resonant converter together within a single, unified framework. The adaptive behaviour of SGO allows it to identify the global peak even when irradiance varies rapidly, while the PSFB resonant stage ensures isolated power transfer, reduced switching losses, and improved operational safety. When combined, these two elements complement each other the MPPT algorithm consistently extracts the available PV power, and the converter maintains high-efficiency regulation over a wide range of operating conditions.

This integrated concept also builds upon the authors’ earlier work on socially inspired MPPT approaches, enabling the present system to remain lightweight, scalable, and suited for practical hardware implementation. Motivated by this gap, the proposed architecture couples an SGO-based MPPT technique with a PSFB resonant converter and investigates their coordinated operation under both uniform and partial-shading scenarios. The findings show improved tracking accuracy, enhanced conversion efficiency, and stronger reliability, thereby addressing the identified gap and offering a practical pathway for efficient PV-powered battery charging in EV applications.

The primary contributions of this research are as follows:

1. An adaptive MPPT scheme based on Social Group Optimization (SGO) is employed to improve power extraction from the PV array, with its robustness demonstrated under various partial-shading patterns.
2. A high-efficiency PSFB Full bridge resonant converter is developed, incorporating soft switching to establish enhanced power delivery.
3. A unified phase-shift control approach is introduced to coordinate MPPT and battery charging, enabling natural ZVS through device capacitances and transformer leakage, which helps lower switching losses.

The structure of the paper is as follows: "SGO algorithm based PSFB for battery charging" section deals with mathematical modelling of the photovoltaic system. The partial shading conditions are critically analysed, highlighting the impact on output characteristics such as multiple maximum power points. To address this the SGO algorithm has been introduced. "Phase shift full bridge resonant converter" section deals with PV fed phase shift full bridge resonant converter with efficient power conversion. The simulation validation and hardware implementation which ensures the efficiency improvement, soft switching characteristics and voltage regulation. In addition, the integration of SGO algorithm with phase shift full bridge resonant converter under partial shading condition provides improved maximum power point tracking and efficient converter performance. "Conclusion" section presents the key findings of the MPPT algorithm and the converter efficiency.

### Effects of shading on photovoltaic arrays

**Fig. 2:**
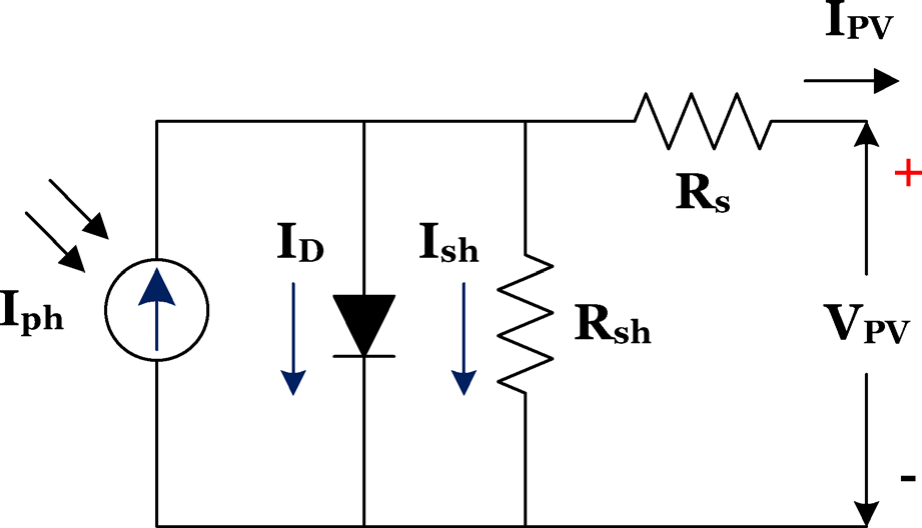
Equivalent circuit of pv cell^19^.

A PV cell’s equivalent circuit is depicted as a current source connected in parallel with leakage elements, represented by a shunt resistance R_sh_. Figure 2 Shows that s single solar cell modelling which should be expandable as a PV array. Voltage is produced by the solar panel as a result of sunlight irradiation and the panel’s temperature. The Eqs. (1–3) are derived from the equivalent circuit and formulated through the diode equation and Kirchoff’s rules.

Here the diode current is considered as equal to short circuit current.

1 $${{\mathrm{I}}_{\mathrm{d}}}={{\mathrm{I}}_{{\mathrm{PV}}}} - \left( {{{\mathrm{e}}^{\frac{{{\mathrm{Vpv}}}}{{ \propto {\mathrm{kT}}}}}} - 1} \right)$$

From the equivalent circuit

2 $${\mathrm{I}_{\mathrm{P}\mathrm{V}}}=\mathrm{I} - {\mathrm{I}_\mathrm{d}}+\frac{{{\mathrm{V}_{\mathrm{p}\mathrm{v}}}+{\mathrm{I}_{\mathrm{p}\mathrm{v}}}{\mathrm{R}_\mathrm{s}}}}{{{\mathrm{R}_{\mathrm{s}\mathrm{h}}}}}$$

3 $$\mathrm{I}={\mathrm{I}_{\mathrm{p}\mathrm{h}}} - {\mathrm{I}_{\mathrm{p}\mathrm{v}}}\left( {{\mathrm{e}^{\frac{{{\mathrm{v}_{\mathrm{p}\mathrm{v}}}}}{{\upalpha \mathrm{k}\mathrm{T}}}}} - 1} \right)+\left( {\frac{{{\mathrm{V}_{\mathrm{p}\mathrm{v}}}+{\mathrm{I}_{\mathrm{p}\mathrm{v}}}{\mathrm{R}_\mathrm{s}}}}{{{\mathrm{R}_{\mathrm{s}\mathrm{h}}}}}} \right)$$

Maximum power refers to the peak instantaneous power determined by the prevailing environmental conditions. It is calculated as the product of voltage and current at that moment, as expressed in the Eq. (4)

4 $${\mathrm{M}_{\mathrm{p}\mathrm{v}}}={\mathrm{V}_{\mathrm{p}\mathrm{v}}} \times {\mathrm{I}_{\mathrm{p}\mathrm{v}}}$$

**Fig. 3:**
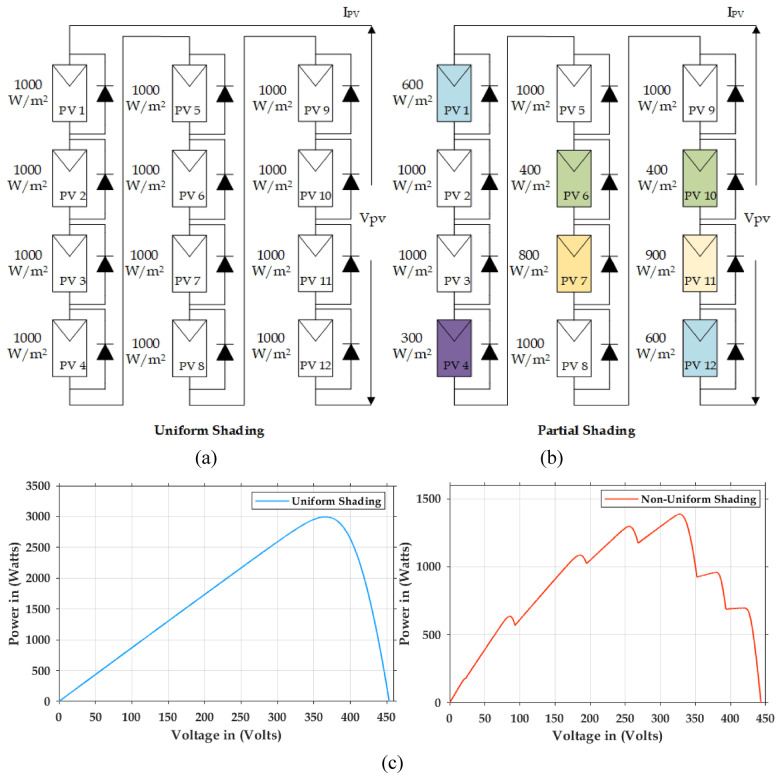
Solar Photovoltaic system under partially shading (**a**) string arrangement for uniform shading (**b**) string arrangement for non-uniform (**c**) shading characteristics analysis for multiple peaks.

Figure 3a illustrates the string arrangement for uniform shading which is providing 3 kW power to the full bridge converter with irradiation of 1000 w/m^2^. Figure 3b shows that the partial shading in PV systems occur due to the hindrances that obstruct the exposure of PV panels to sunlight. These obstructions may happen due to natural blockages like trees, buildings etc. or due to man-made ones like chimneys, utility poles etc. or even due to weather and environmental disturbances like clouds, dust, debris etc. Due to the shading of even fewer cells in a panel, the net output power decreases. The cells with shading acts as a resistive load and it do emit heat instead of electric power. In a standard PV panel, the cells are connected in series, and shaded cells generate less current. This reduced current becomes the overall current, leading to a decrease in the total power output. Therefore, there may be 50% of power loss even if there is 10% of shading. Bypass diodes are prudent choice for mitigating the impact of shading. These diodes facilitate the blocked current of the shaded cells to get bypassed and thereby aiding to have better efficiency levels for the entire solar array. This ensures more consistent energy production, especially in environments prone to partial shading. When bypass diodes are used, a key issue is the formation of multiple power peaks in the current-voltage (I–V) and P–V curves. Under uniform sunlight, the P–V curve has a single, well-defined maximum power point (MPP). However, if part of the PV array is shaded, the bypass diodes redirect current around the shaded panels, resulting in multiple power peaks, one corresponding to the unshaded area and another to the shaded area.

Figure 3c Depicts the string configuration of a partially shaded PV array, where the I–V and P–V curves exhibit multiple power peaks. Traditional MPPT algorithms, which scan the P–V curve to locate the maximum power point, often get stuck at local peaks, resulting in significantly reduced power output. To overcome this limitation and ensure the delivery of maximum global power, this study employs an intelligent SGO based global search algorithm.

## SGO algorithm based PSFB for battery charging

### Social group optimization

The SGO algorithm makes most out of the individual knowledge of participants in a group and achieve the goal. The members in a group, based on their competencies can be named as leaders, followers^21^.The leaders share their experience, and the followers and learners acquire the knowledge shared and with the experience they gain in the search process move towards the objective. The SGO consists of two phases: (i) Improving Phase (ii) Acquiring Phase. The first phase intends to diversify of the search by different regions of the solution space. This phase investigates the search space to identify potential solutions. The second phase is used to utilize the regions of the search space. Individuals share and leverage the collective knowledge within their social groups to concentrate their efforts on areas with potential optimal solutions.

#### Enhancement phase

In this phase, the top performing candidate of each social group, referred to as the global optimum (g_opt_), shares knowledge with other members of the group. This knowledge sharing process enhances the performance of the participating members. The objective function for maximization is defined as g_opt_ = max {F_i_ | i = 1, 2, …., M}. where M represents the total number of candidates in the group, and F_i_ is the fitness value of the i-th candidate. Additionally, during each iteration of this phase, knowledge is exchanged and updated among the candidates, as represented by Eq. (5).

5 $${\mathrm{Y}}_{{{\mathrm{new}},{\mathrm{j}}}}^{{\mathrm{t}}}={{{\upalpha}}} \cdot {\mathrm{Y}}_{{{\mathrm{old}},{\mathrm{j}}}}^{{\mathrm{t}}}+{{{\upbeta}}} \cdot {{{\upxi}}} \cdot \left( {{\mathrm{g}}_{{{\mathrm{opt}}}}^{{{\mathrm{t}}}} - {\mathrm{Y}}_{{{\mathrm{old}},{\mathrm{j}}}}^{{{\mathrm{t}}}}} \right)$$

where ξ is random selection, $\:{\mathrm{Y}\:}_{\mathrm{n}\mathrm{e}\mathrm{w},\mathrm{j}}^{\mathrm{t}}$ is the Updated new position, $\:{\upbeta\:}$ is the learning factor, $\:{\mathrm{g}}_{\:\mathrm{o}\mathrm{p}\mathrm{t}}^{\:\mathrm{t}}$ is the current best solution in the group at iteration t, $\:{\mathrm{Y}}_{\mathrm{o}\mathrm{l}\mathrm{d},\mathrm{j}}^{\mathrm{t}}$ previous position. After calculating $\:{\mathrm{Y}\:}_{\mathrm{n}\mathrm{e}\mathrm{w},\mathrm{j}}^{\mathrm{t}}$ its fitness is evaluated. If the new state performs better than the old one in terms of the objective function, the update is accepted.

#### Knowledge enhancement phase

**Fig. 4:**
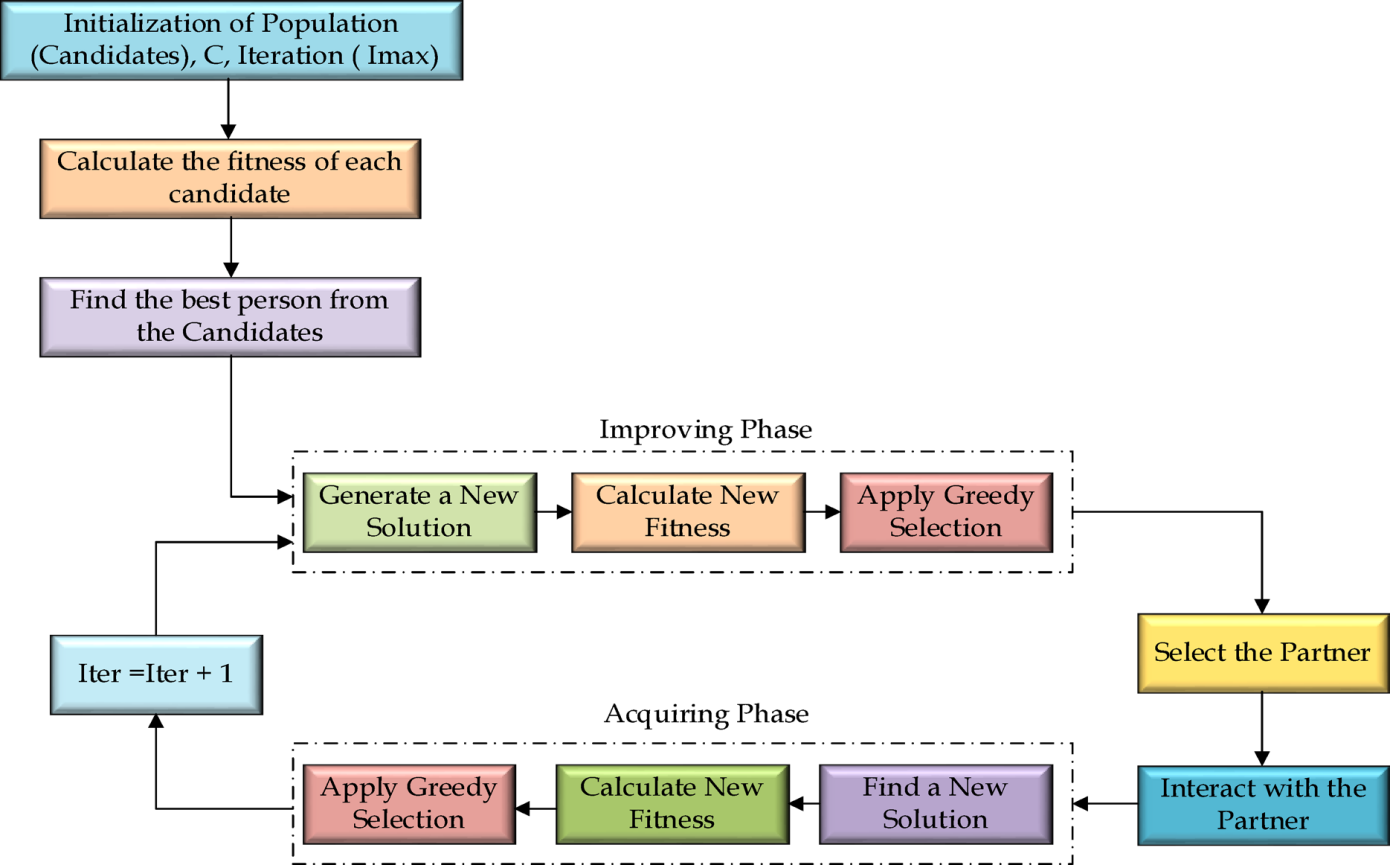
Social group optimization with individual group.

During this phase, each group member gains knowledge from the most knowledgeable individual and engages in random interactions with other members. Candidates acquire new insights both from one another and from the top performer, referred to as g_best_ if another individual surpasses g_best_ in knowledge, they will take the position of the best candidate, as illustrated in Fig. 4. the updated new knowledge valu can be calculated by Eqs. (6) and (7).

If the selected member (Q_r_) has lower knowledge than the current candidate (Q_j_)

6 $${\mathrm{Z}}_{{{\mathrm{new}},{\mathrm{j}}}}^{{\mathrm{k}}}={\mathrm{Z}}_{{{\mathrm{old}},{\mathrm{j}}}}^{{\mathrm{k}}}+{\varphi _1}\left( {{\mathrm{Z}}_{{\mathrm{j}}}^{{{\mathrm{k}}}} - {\mathrm{Z}}_{{{\mathrm{j}}}}^{{{\mathrm{k}}}}} \right)+{\varphi _2}\left( {{\mathrm{g}}_{{{\mathrm{best}}}}^{{{\mathrm{k}}}} - {\mathrm{Z}}_{{{\mathrm{j}}}}^{{{\mathrm{k}}}}} \right)$$

If the selected member Q_r_ has greater knowledge than the current candidate Q_j_

7 $${\mathrm{Z}}_{{{\mathrm{new}},{\mathrm{j}}}}^{{\mathrm{k}}}={\mathrm{Z}}_{{{\mathrm{old}},{\mathrm{j}}}}^{{\mathrm{k}}} - {\varphi _1}\left( {{\mathrm{Z}}_{{\mathrm{j}}}^{{{\mathrm{k}}}} - {\mathrm{Z}}_{{{\mathrm{j}}}}^{{{\mathrm{k}}}}} \right)+{\varphi _2}\left( {{\mathrm{g}}_{{{\mathrm{best}}}}^{{{\mathrm{k}}}} - {\mathrm{Z}}_{{{\mathrm{j}}}}^{{{\mathrm{k}}}}} \right)$$

where,

Q_j_ = The current candidate.

Q_r_ = A randomly selected group member.

$\:{\mathrm{Z}\:}_{\mathrm{n}\mathrm{e}\mathrm{w},\mathrm{j}}^{\mathrm{k}}$ = The updated new knowledge value of candidate Q_j_ in the k^th^ dimension.

$\:{\mathrm{Z}}_{\mathrm{o}\mathrm{l}\mathrm{d},\mathrm{j}}^{\mathrm{k}}$ = The previous value of candidate Q_j_ in the k^th^ dimension.

$\:{\mathrm{g}}_{\mathrm{b}\mathrm{e}\mathrm{s}\mathrm{t}}^{\:\mathrm{k}}$ = Best knowledge in the group.

$\:{\phi\:}_{1}$ = Learning coefficient component.

$\:{\phi\:}_{2}$ = Global learning coefficient.

k = Dimension index.

The process begins by randomly initializing the duty cycle of the PSFB within a defined range, constrained by the open -circuit voltage (V_oc_) and short-circuit current (I_sc_) of the PV system. By using this initial duty cycle, the power output of the PV system is computed. The duty cycle corresponding to the highest power output is identified as the leader, while the remaining duty cycles are categorized as learners. To achieve maximum power point tracking, the search mechanism is updated iteratively, with solutions progressing toward the leader. The duty cycles represent the participating members in this optimization framework.

#### Exploration phase

In the exploration phase, candidates moved based on their previous positions and the influence of the best-performing member. This phase is represented as Eq. (8)

8 $${\mathrm{D}}_{{{\mathrm{new}},{\mathrm{j}}}}^{{\mathrm{k}}}={{{\upgamma}}} \cdot {\mathrm{D}}_{{{\mathrm{old}},{\mathrm{j}}}}^{{\mathrm{k}}}+{{{\uprho}}} \cdot \left( {{\mathrm{G}}_{{{\mathrm{best}}}}^{{{\mathrm{k}}}} - {\mathrm{D}}_{{{\mathrm{old}},{\mathrm{j}}}}^{{{\mathrm{k}}}}} \right)$$

$\:{\mathrm{D}}_{\mathrm{n}\mathrm{e}\mathrm{w},\mathrm{j}}^{\mathrm{k}}$—Updated duty cycle of the candidate j at iteration k.

$\:{\mathrm{D}}_{\mathrm{o}\mathrm{l}\mathrm{d},\mathrm{j}}^{\mathrm{k}}$—Previous duty cycle of candidate j.

$\:{\upgamma\:}$—Self adjustment factor in the range (0,1).

ρ—Random coefficient to introduce variability from (0,1).

$\:{\mathrm{G}}_{\mathrm{b}\mathrm{e}\mathrm{s}\mathrm{t}}^{\:\mathrm{k}}$—Current best solution in the group.

#### Exploitation phase

The candidates further refine their solutions based on comparisons with randomly selected alternatives. This as follows in the Eqs. (9) and (10).

If $\:{\mathrm{D}}_{\mathrm{n}\mathrm{e}\mathrm{w},\mathrm{j}}^{\mathrm{k}}$ performs better than $\:{\mathrm{D}}_{\mathrm{r}\mathrm{a}\mathrm{n},\mathrm{j}}^{\mathrm{k}}$:

9 $${\mathrm{D}}_{{{\mathrm{new}},{\mathrm{j}}}}^{{\mathrm{k}}}={\mathrm{D}}_{{{\mathrm{old}},{\mathrm{j}}}}^{{\mathrm{k}}}+{{{{\upsigma}}}_1}\left( {{\mathrm{D}}_{{\mathrm{j}}}^{{{\mathrm{k}}}} - {\mathrm{D}}_{{{\mathrm{ran}},{\mathrm{j}}}}^{{{\mathrm{k}}}}} \right)+{{{{\upsigma}}}_2}\left( {{\mathrm{g}}_{{{\mathrm{best}}}}^{{{\mathrm{k}}}} - {\mathrm{D}}_{{{\mathrm{j}}}}^{{{\mathrm{k}}}}} \right)$$

If $\:{\mathrm{D}}_{\mathrm{r}\mathrm{a}\mathrm{n},\mathrm{j}}^{\mathrm{k}}$ performs better than $\:{\mathrm{D}}_{\mathrm{n}\mathrm{e}\mathrm{w},\mathrm{j}}^{\mathrm{k}}$

10 $${\mathrm{D}}_{{{\mathrm{new}},{\mathrm{j}}}}^{{\mathrm{k}}}={\mathrm{D}}_{{{\mathrm{old}},{\mathrm{j}}}}^{{\mathrm{k}}} - {{{{\upsigma}}}_1}\left( {{\mathrm{D}}_{{\mathrm{j}}}^{{{\mathrm{k}}}} - {\mathrm{D}}_{{{\mathrm{ran}},{\mathrm{j}}}}^{{{\mathrm{k}}}}} \right)+{{{{\upsigma}}}_2}\left( {{\mathrm{g}}_{{{\mathrm{best}}}}^{{{\mathrm{k}}}} - {\mathrm{D}}_{{{\mathrm{j}}}}^{{{\mathrm{k}}}}} \right)$$

$\:{\mathrm{D}}_{\:\mathrm{r}\mathrm{a}\mathrm{n},\mathrm{j}}^{\:\mathrm{k}}$—Duty cycle of a randomly selected candidate.

$\:{{\upsigma\:}}_{1}\:,$ $\:{{\upsigma\:}}_{2}$—Random scaling influencing local and global adjustments.

$\:{\mathrm{g}}_{\mathrm{b}\mathrm{e}\mathrm{s}\mathrm{t}}^{\:\mathrm{k}}$—Influencing of the best-performing duty cycle.

**Fig. 5:**
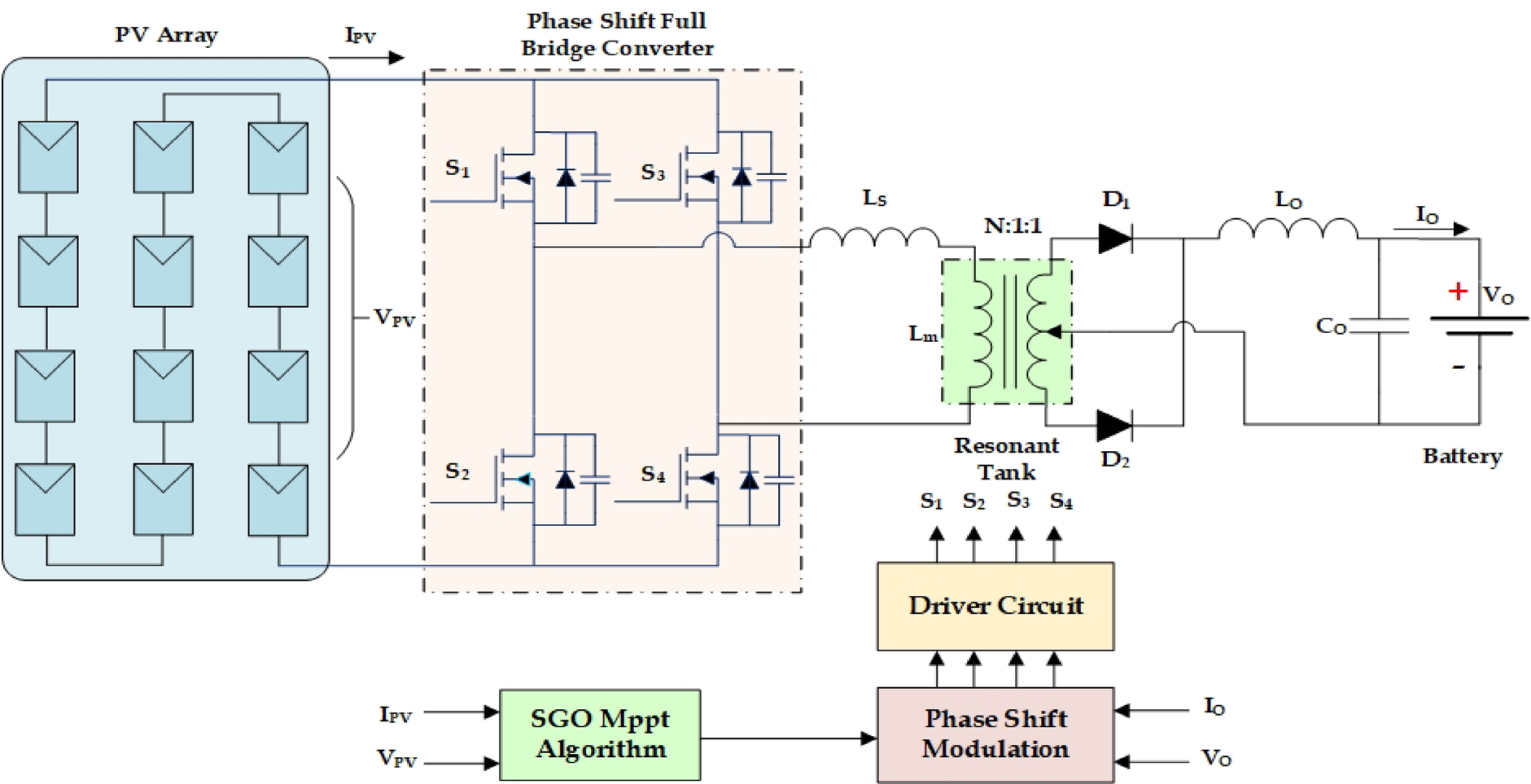
PV aided phase shift full bridge resonant converter.

The partially shaded PV array is optimized through SGO MPPT, and it is hybridized with the PSFB converter for battery charging in EV bays. This section details the SGO MPPT, full bridge design and resonant operation and phase-shift modulation. Figure 5. presents PV aided charging system through the full bridge resonant converter and hybrid SGO phase shift control scheme. The PV system consists of 12 series connected modules of 275 W yielding a voltage of 469 V (12 × 39 V) at open circuit and 390 V (12 × 32.5 V) at maximum power.

## Phase shift full bridge resonant converter

The phase shift full bridge resonant converter is used to regulate the output power while frequency is constant which implies to reduce the magnetic design. The converter achieves zero voltage switching (ZVS) using transformer leakage inductance and MOSFET capacitance, reducing switching losses and improving efficiency. The PSFB converter can be used for wide input and output voltage and provides a fast transient response which is suitable for dynamic loads. At light loads, it maintains good performance through burst mode control. The PSFB converter is having some additional characteristics such as (i) galvanic isolation is provided by the high frequency transformer which ensures the safety and ground loop interference (ii) smooth control of power flow is achieved by modulating the phase difference between the two inverter legs, eliminating the need of duty cycle variation. (iii) reduced switching stress and EMI due to zero voltage switching in the switch which cause smaller magnetic and filter components. (iv) flexible transformer ratio allows adaptation to a wide range of input PV voltages and battery charging voltages. (v) compatibility with digital control platforms, enabling seamless integration with MPPT algorithms and closed loop voltage and current regulation. The bridge converter consists of four switches S_1_, S_2_, S_3_, and S_4_ on the primary side of the high-frequency transformer, with a centre taped rectifier connected on the secondary side. The battery pack is rated at 3.3 kW with 48 V as the operating voltage. The phase shift full bridge resonant topology is employed here to ensure efficient power delivery. The phase shift controller ensures good voltage regulation, achieves ZVS, and provides better efficiency with reduced power losses. The resonant frequency (f_r_) of the tank circuit is determined by the specified maximum transition time and the requirement for stored inductive energy. The components of this tank circuit consist of the resonant inductor (Lr) and capacitor (Cr) which are derived from the output capacitors of the two switches. The resonant tank parameters are calculated using the Eqs. (11–14).

11 $${\text{The resonant frequency}}\;{\mathrm{f}_\mathrm{r}}=\frac{1}{{\sqrt {{\mathrm{L}_\mathrm{r}} \times {\mathrm{C}_\mathrm{r}}} }}$$

12 $${\text{Maximum switch transition time}}=\frac{\uppi }{{2{\mathrm{f}_\mathrm{r}}}}$$

The resonant capacitance is

13 $${\mathrm{C}_\mathrm{r}}=\frac{8}{3} \times {\mathrm{C}_{\mathrm{o}\mathrm{s}\mathrm{s}}} \times {\mathrm{C}_\mathrm{p}}$$

The resonant inductance is

14 $${\mathrm{L}_\mathrm{r}}=\frac{1}{{\mathrm{f}_{\mathrm{r}}^{2} \times {\mathrm{C}_\mathrm{r}}}}$$

**Table 1:** Modes of operation of PSFB

Interval	S_1_	S_2_	S_3_	S_4_	Description
(t_0_ to t_1_)	ON	OFF	OFF	OFF	Right leg transition
(t_1_ to t_2_)	ON	OFF	ON	OFF	Free wheeling interval
(t_2_ to t_3_)	OFF	OFF	ON	OFF	Left leg transition
(t_3_ to t_4_)	OFF	ON	ON	OFF	Power transfer

**Phase 1 (0 to t**_**1**_ **)**

**Fig. 6:**
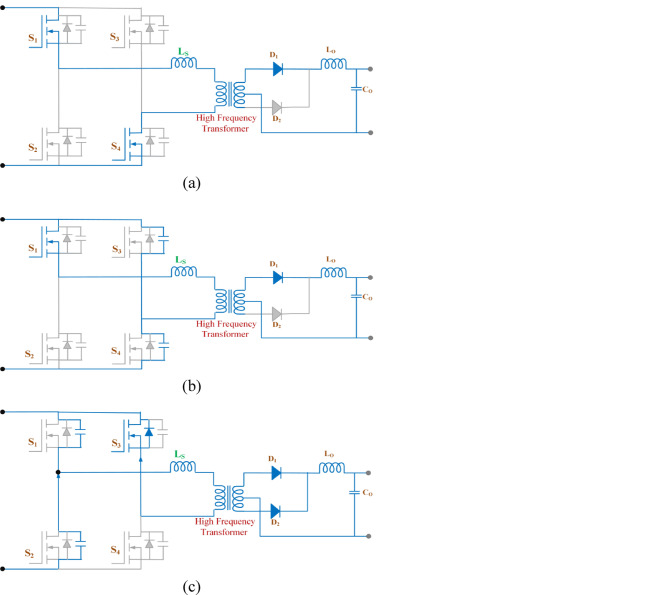

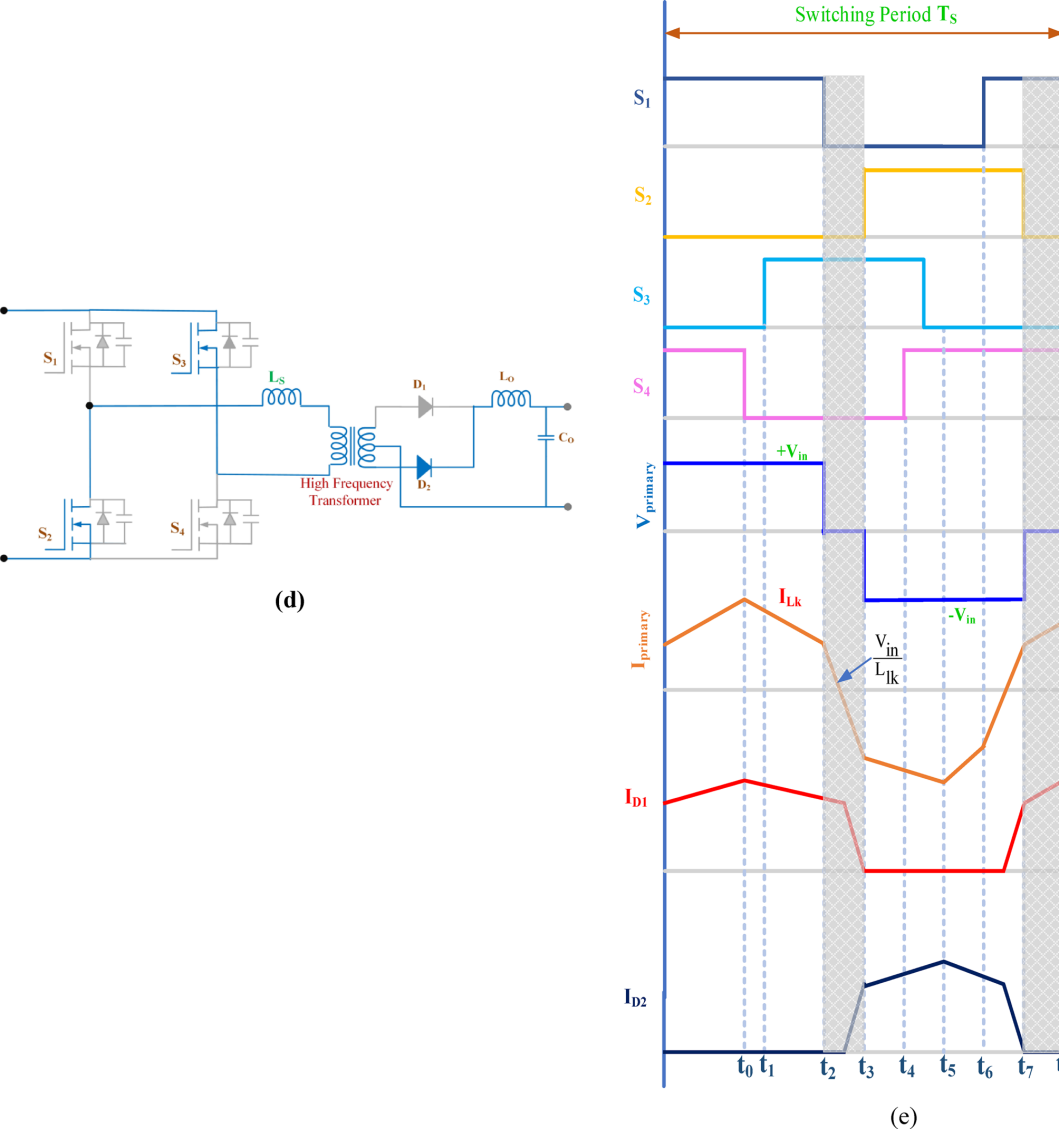
Converter modes of operation (**a**) Phase 1 (0 to t_1_), (**b**) Phase 2 (t_1_ to t_2_), (**c**) Phase 3 (t_2_ to t_3_), (**d**) Phase 4 (t_3_ to t_4_) (**e**) Key waveform of PSFB.

Figure 6. illustrates modes of operation of PSFB converter. At the start, at time t = 0, the primary side current is zero. As time progresses from 0 to t_1_. Switch S_1_ begins conducting, as illustrated in Fig. 6a. During this initial phase, the primary current remains constant due to resonance, which is determined by the transformer leakage inductance (I_lk_). When diode D_1_ starts conducting, energy is transferred from primary to secondary side. Following this, switch S_4_ is turned off, causing the transformer to enter a short-circuit state, and the voltage across the transformer drops to zero. The parasitic output capacitance (C_oss_) of S_4_ is charged, while the C_oss_ of switch S_3_ discharges.

Based on the phase 1 equivalent circuit of the PSFB^27^, the primary current and voltage across the circuit are calculated as per Eqs. (15) and (16)

15 $$\mathrm{v}(\mathrm{t})={\mathrm{V}_{\mathrm{i}\mathrm{n}}} - \frac{1}{{2{\mathrm{C}_{\mathrm{o}\mathrm{s}\mathrm{s}}}+\frac{{4{\mathrm{C}_{\mathrm{j}\mathrm{d}}}}}{\mathrm{n}}}}{\mathrm{I}_{\mathrm{p}\mathrm{p}}}(\mathrm{t} - {\mathrm{t}_0}) - \frac{{\frac{{4{\mathrm{C}_{\mathrm{j}\mathrm{d}}}}}{\mathrm{n}}}}{{2\upomega {\mathrm{C}_{\mathrm{o}\mathrm{s}\mathrm{s}}}\left( {2{\mathrm{C}_{\mathrm{o}\mathrm{s}\mathrm{s}}}+\frac{{4{\mathrm{C}_{\mathrm{j}\mathrm{d}}}}}{\mathrm{n}}} \right)}}{\mathrm{I}_{\mathrm{p}\mathrm{p}}}\mathrm{s}\mathrm{i}\mathrm{n}\upomega (\mathrm{t} - \mathrm{t}{}_{0})$$

16 $${\mathrm{i}_\mathrm{p}}(\mathrm{t})=\frac{{\frac{{4{\mathrm{C}_{\mathrm{j}\mathrm{d}}}}}{\mathrm{n}}{\mathrm{I}_{\mathrm{p}\mathrm{p}}}}}{{2{\mathrm{C}_{\mathrm{o}\mathrm{s}\mathrm{s}}}+\frac{{4{\mathrm{C}_{\mathrm{j}\mathrm{d}}}}}{\mathrm{n}}}}\left( {\mathrm{c}\mathrm{o}\mathrm{s}\upomega (t - {\mathrm{t}_0})+\frac{{2{\mathrm{C}_{\mathrm{o}\mathrm{s}\mathrm{s}}}}}{{\frac{{4{\mathrm{C}_{\mathrm{j}\mathrm{d}}}}}{\mathrm{n}}}}} \right)$$

**Phase 2 (t**_**1**_ **to t**_**2**_**)**

At instant t_1_, when switches S_1_ and S_4_ are turned off, the inductor current (I_L_) discharges the parasitic capacitances (C_oss_) of S_1_ and S_4_, while simultaneously charging the capacitances of S_2_ and S_3_ in preparation for the next switching transition as shown in Fig. 6b.

The current and voltage of the primary can be expressed in Eqs. (17) and (18)

17 $$\mathrm{v}(\mathrm{t})={\mathrm{I}_{\mathrm{p}\mathrm{p}}}\sqrt {\frac{{{\mathrm{L}_{\mathrm{l}\mathrm{k}}}}}{{2{\mathrm{C}_{\mathrm{o}\mathrm{s}\mathrm{s}}}}}} \mathrm{s}\mathrm{i}\mathrm{n}\left( {\frac{1}{{\sqrt {2{\mathrm{L}_{\mathrm{l}\mathrm{k}}}} {\mathrm{C}_{\mathrm{o}\mathrm{s}\mathrm{s}}}}}(\mathrm{t} - {\mathrm{t}_2})} \right)$$

18 $${\mathrm{i}_\mathrm{p}}(\mathrm{t})={\mathrm{I}_{\mathrm{p}\mathrm{p}}}\mathrm{c}\mathrm{o}\mathrm{s}\left( {\frac{1}{{\sqrt {2{\mathrm{I}_{\mathrm{l}\mathrm{k}}}\mathrm{C}{}_{{\mathrm{o}\mathrm{s}\mathrm{s}}}} }}(\mathrm{t} - {\mathrm{t}_0})} \right)$$

**Phase 3 (t**_**2**_ **to t**_**3**_**)**

When switches S_1_ and S_4_ turn off, the diagonal switches S_2_ and S_3_ will begin conducting. The current path on the primary side will shift, passing through the parasitic capacitance (C_oss_) of switch S_1_. This current path helps raise and lower the voltage across switch S_2_, enabling it to transition under ZVS conditions. The body diode of S_2_ temporarily conducts to clamp voltage, maintaining control over the primary current. Once S_2_ begins to turn on, switch S_3_ (already conducting) will allow power transfer to proceed through the transformer as shown in Fig. 6c.

From Eqs. (19) and (20) the current through the diode rectifier D_1_ and D_2_ is

19 $${\mathrm{i}_{\mathrm{D}1}}=\frac{{{\mathrm{I}_\mathrm{o}}}}{2}\left( {1+\mathrm{c}\mathrm{o}\mathrm{s}\frac{\mathrm{n}}{{\sqrt {{\mathrm{L}_\mathrm{r}}} {\mathrm{C}_\mathrm{r}}}}(\mathrm{t} - {\mathrm{t}_3})} \right)$$

20 $${\mathrm{i}_{\mathrm{D}2}}=\frac{{{\mathrm{I}_\mathrm{o}}}}{2}\left( {1 - \mathrm{c}\mathrm{o}\mathrm{s}\frac{\mathrm{n}}{{\sqrt {{\mathrm{L}_\mathrm{r}}} {\mathrm{C}_\mathrm{r}}}}(\mathrm{t} - {\mathrm{t}_3})} \right)$$

**Phase 4 (t**_**3**_ **< t < t**_**4**_**)**

Now, the phase-shifted cycle is now equivalent to a standard square wave conversion. After switch S_4_ turns off, the cycle repeats from the initial stage. Switch S_3_ will remain off, but current flows through the parasitic capacitance, increasing the input voltage from zero to the source voltage as shown in Fig. 6d. Key waveform of PSFB is shown in Fig. 6e. All these modes of operation are presented in Table 1.

### Design considerations

To optimize voltage regulation and efficiency, it is crucial to carefully select key parameters, including the parasitic capacitance of the switches, the shim inductor, the transformer core, and its magnetising inductance. On the secondary side, critical considerations include the use of a half- wave rectifier and the design of the output filter. In a PSFB topology, the transformer plays avital role in transferring energy from the PV input to the battery charging output through magnetic coupling. It facilitates resonant operation by managing the phase shift between switching pulses and achieves voltage transformation between the primary and secondary sides based on the turn’s ratio.

The turns ratio is calculated by using Eqs. (21)–(22) and from the magnetising inductance which is mentioned in Eq. (23)

21 $$\mathrm{n}=\frac{{{\mathrm{N}_\mathrm{p}}}}{{{\mathrm{N}_\mathrm{s}}}}$$

22 $$\mathrm{n}=\frac{{\left( {{\mathrm{V}_{\mathrm{i}\mathrm{n}\_\mathrm{m}\mathrm{i}\mathrm{n}}} - 2 \times {\mathrm{V}_{\mathrm{R}\mathrm{D}\mathrm{S}\_\mathrm{O}\mathrm{N}}}} \right) \times {\mathrm{D}_{\mathrm{m}\mathrm{a}\mathrm{x}}}}}{{{\mathrm{V}_\mathrm{o}}+{\mathrm{V}_{\mathrm{R}\mathrm{D}\mathrm{S}\_\mathrm{O}\mathrm{N}}}}}$$

23 $$\Delta {\mathrm{I}_{\mathrm{L}\_\mathrm{O}\mathrm{u}\mathrm{t}}}=\frac{{{\mathrm{P}_\mathrm{o}} \times 0.2}}{{{\mathrm{V}_\mathrm{o}}}}\left( {{\text{The peak}} - {\mathrm{to}} - {\text{peak current ripple is set to 2}}0\% } \right)$$

The PSFB operates in voltage mode control for low values and in peak current mode control in for high values, magnetizing inductance (L_mag_) can be calculated by Eq. (24)

24 $${\mathrm{L}_{\mathrm{m}\mathrm{a}\mathrm{g}}} \geq \frac{{\frac{{{\mathrm{V}_{\mathrm{i}\mathrm{n}}}(1 - \mathrm{D})}}{{\Delta {\mathrm{I}_{\mathrm{L}\_\mathrm{o}\mathrm{u}\mathrm{t}}} \times 0.5 \times {\mathrm{f}_{\mathrm{s}\mathrm{w}}}}}}}{\mathrm{n}}$$

The transformer primary and secondary current ca. be calculated by Eqs. (25)-(26)

25 $${\mathrm{I}_{\mathrm{r}\mathrm{m}\mathrm{s}\_\mathrm{s}}}=\frac{{\Delta {\mathrm{I}_{\mathrm{L}\_\mathrm{O}\mathrm{u}\mathrm{t}}}}}{2}\sqrt {\left( {\frac{{1 - {\mathrm{D}_{\mathrm{m}\mathrm{a}\mathrm{x}}}}}{6}} \right)}$$

26 $${\mathrm{I}_{\mathrm{r}\mathrm{m}\mathrm{s}\_\mathrm{p}}}=\sqrt {{\mathrm{D}_{\mathrm{m}\mathrm{a}\mathrm{x}}}\left( {{\mathrm{I}_{\mathrm{p}\mathrm{p}}} \times {\mathrm{I}_{\mathrm{m}\mathrm{p}}}+\frac{{{{({\mathrm{I}_{\mathrm{p}\mathrm{p}}} - {\mathrm{I}_{\mathrm{m}\mathrm{p}}})}^2}}}{3}} \right)}$$

To maintain the continuous current the inductor has been selected and it reduces the electromagnetic interference, and it helps to improve the efficiency. The output inductor and capacitor can be calculated by Eqs. (27) and (29).

27 $${\mathrm{L}_\mathrm{o}}=\frac{{{\mathrm{V}_\mathrm{o}} \times (1 - \mathrm{D})}}{{2 \times {\mathrm{f}_{\mathrm{s}\mathrm{w}}} \times \Delta {\mathrm{I}_{\mathrm{L}\_\mathrm{O}\mathrm{u}\mathrm{t}}}}}$$

28 $${\mathrm{t}_{\mathrm{H}\mathrm{U}}}=\frac{{\frac{{{\mathrm{L}_\mathrm{o}} \times {\mathrm{P}_\mathrm{o}} \times 0.9}}{{{\mathrm{V}_\mathrm{o}}}}}}{{{\mathrm{V}_\mathrm{o}}}}\;{\text{The time takes }}{{\mathrm{L}}_{\mathrm{o}}}{\text{is to change 9}}0\% {\text{ of full load}}$$

The transient voltage is selected for 10% transient voltage (V_t_)

29 $${\mathrm{C}_\mathrm{o}} \geq \frac{{\frac{{{\mathrm{P}_\mathrm{o}} \times 0.9 \times {\mathrm{t}_{\mathrm{H}\mathrm{U}}}}}{{{\mathrm{V}_\mathrm{o}}}}}}{{{\mathrm{V}_\mathrm{t}} \times 0.1}}$$

The selection of shim inductor is based on the energy required to achieve ZVS in primary side and based on the selection of parasitic capacitance of switch. The minimum value of the shim inductor can be calculated by Eq. (30). Circuit parameters and their corresponding values are given in Table 2)

30 $${\mathrm{L}_\mathrm{s}} \geq \left( {2 \times {\mathrm{C}_{\mathrm{o}\mathrm{s}\mathrm{s}}}} \right)\frac{{{\mathrm{V}_{\mathrm{i}\mathrm{n}}}}}{{\frac{{{\mathrm{I}_{\mathrm{p}\mathrm{p}}}}}{2} - \frac{{\Delta {\mathrm{I}_{\mathrm{L}\_\mathrm{o}\mathrm{u}\mathrm{t}}}}}{{2\mathrm{n}}}}} - {\mathrm{L}_{\mathrm{l}\mathrm{k}}}$$

**Table 2:** Specification of design procedure.

Symbol	Parameter
Input voltage, V_in_	400 V
Maximum input voltage, V_in_Min_ -V_in_Max_	100–366 V
Output voltage V_o_	48 V
Output power, P_o_	3 kW
Switching frequency, f_sw_	100 kHz
Shim inductor, L_s_	16 µH
Output parasitic capacitance, C_oss_	100 pF (V_ds_= 400 V)
Output inductance, L_o_	20.8 µH
Output capacitance, C_o_	325.52 µF

### Simulation results and discussions

**Fig. 7:**
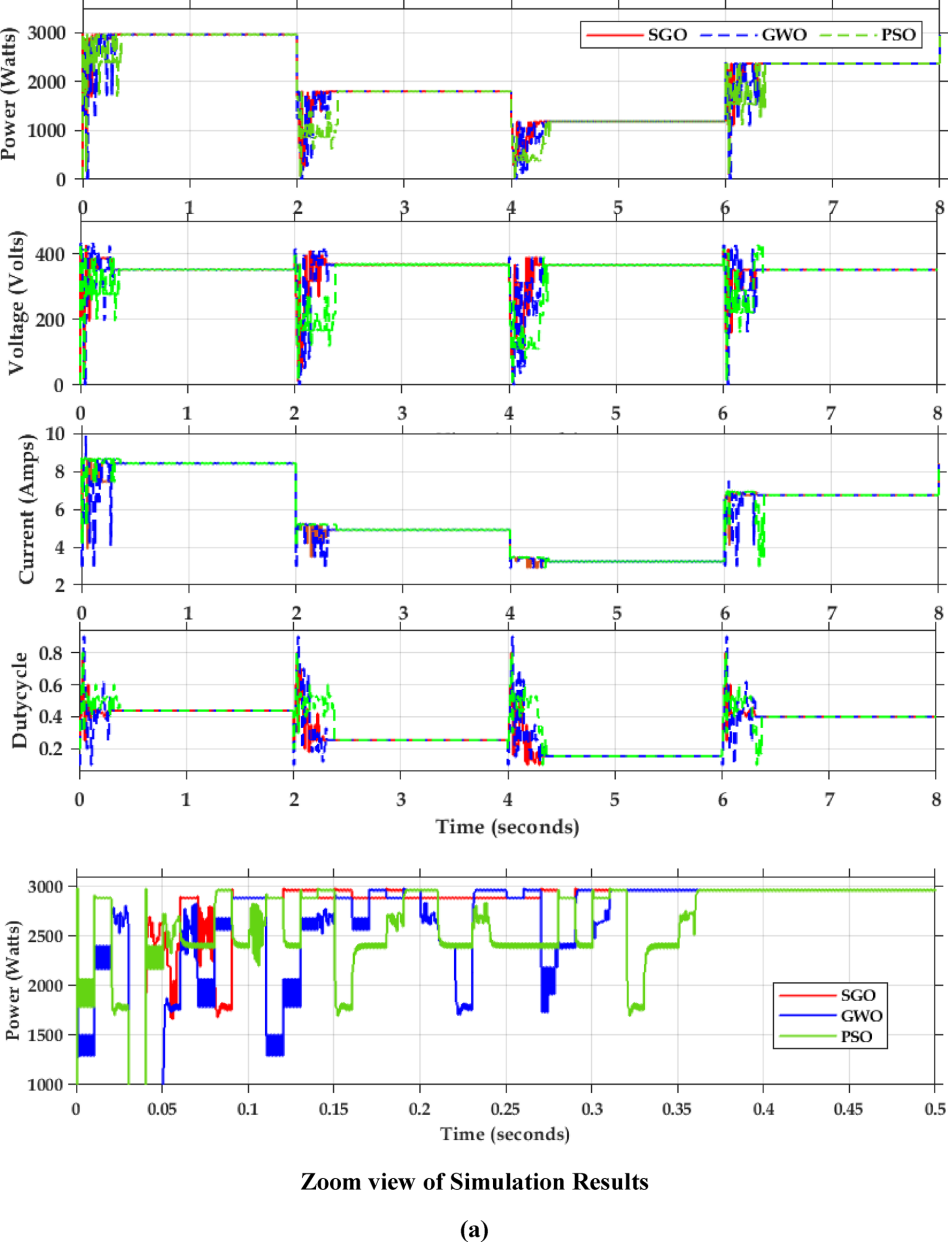

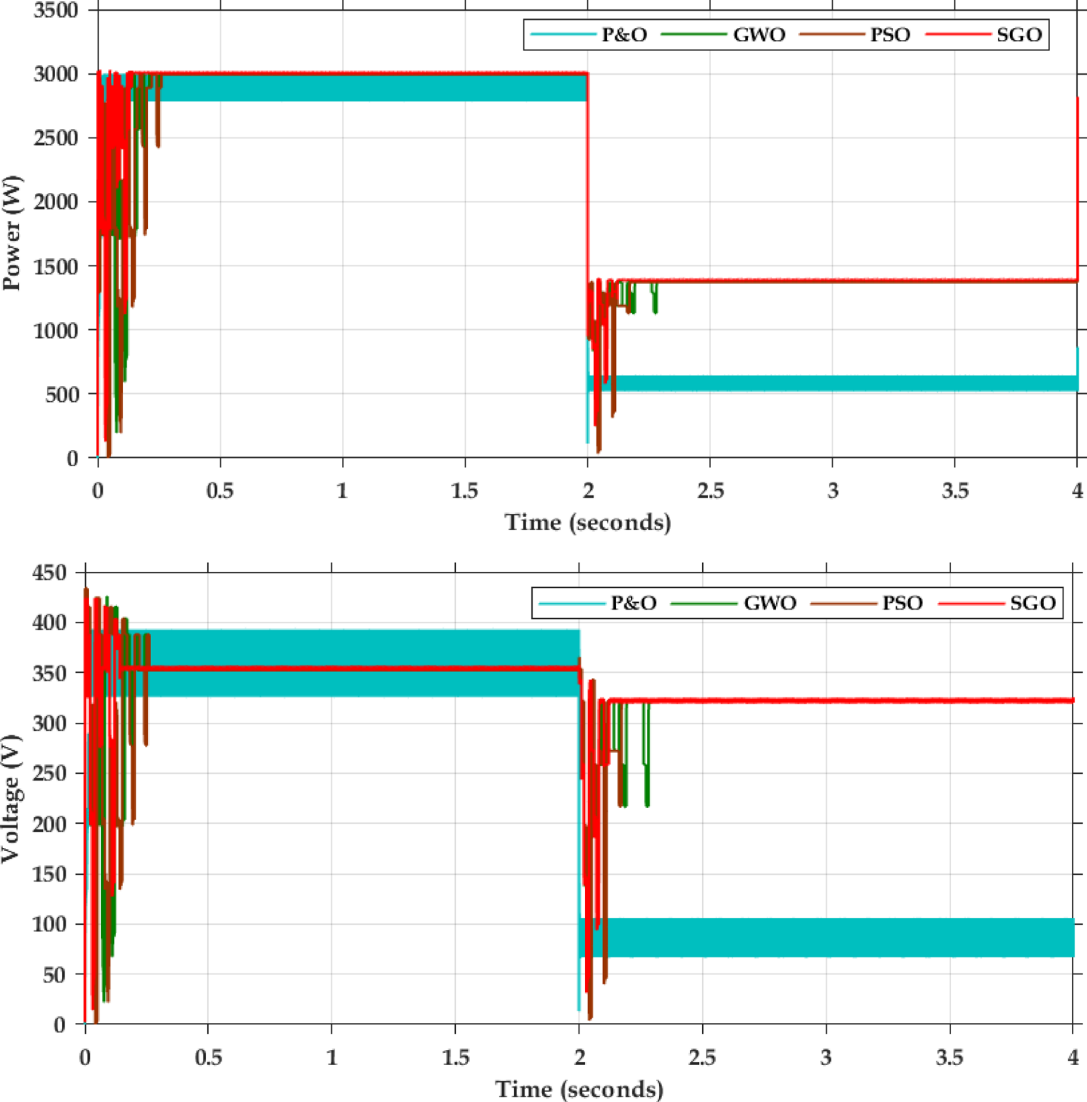

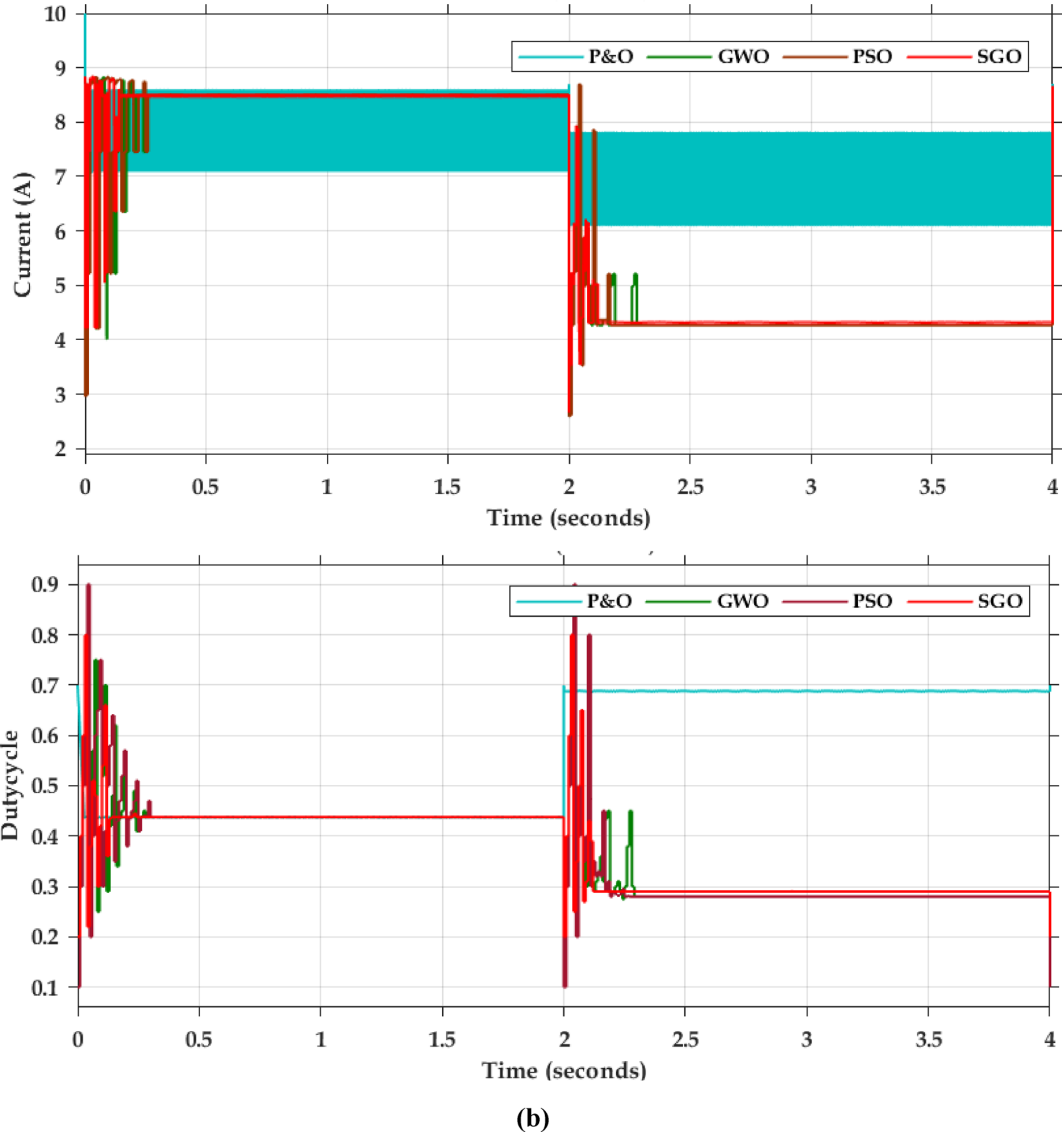
Simulation results of MPPT (**a**) Simulation result of partial shading pattern (**b**) simulation result of dynamic shading.

**Table 3:** MPPT tuning parameters.

MPPT method	Tuning parameters	No of populations	Maximum no of iterations
SGO	$\:{{\upsigma\:}}_{1}\:=\:0.1,\:{{\upsigma\:}}_{2}\:=\:0.9$	5	100
GWO	A and C - random numbers	5	100
PSO	C1max = C2max = 2, C1min = C2min = 1, Wmax = 1, Wmin = 0.1	5	100

**Table 4:** Comparison of MPPT algorithms.

Cases	Algorithms	Iterations	GMPP (W)	Power tracked (W)	Convergence time (s)	Efficiency
Uniform shading	SGO	8	3000	2998	0.14	99.93
	GWO	11		2988	0.25	99.60
	PSO	14		2985	0.26	99.50
	P&O	–		2850	0.05	95.00
Partial shading	SGO	10	1395	1393	0.16	99.85
	GWO	13		1373	0.20	98.42
	PSO	15		1372	0.28	98.35
	P&O	–		550	–	39.42

**Table 5:** Comparison of PSFB efficiency under partially shading condition.

Case	Algorithms	Maximum PV power	PSFB output power	Efficiency
Partial shading	SGO	1393	1261	90.6
	GWO	1373	1243.21	90.5
	PSO	1372	1242.31	90.5
	P&O	550	498.02	90.5

**Fig. 8:**
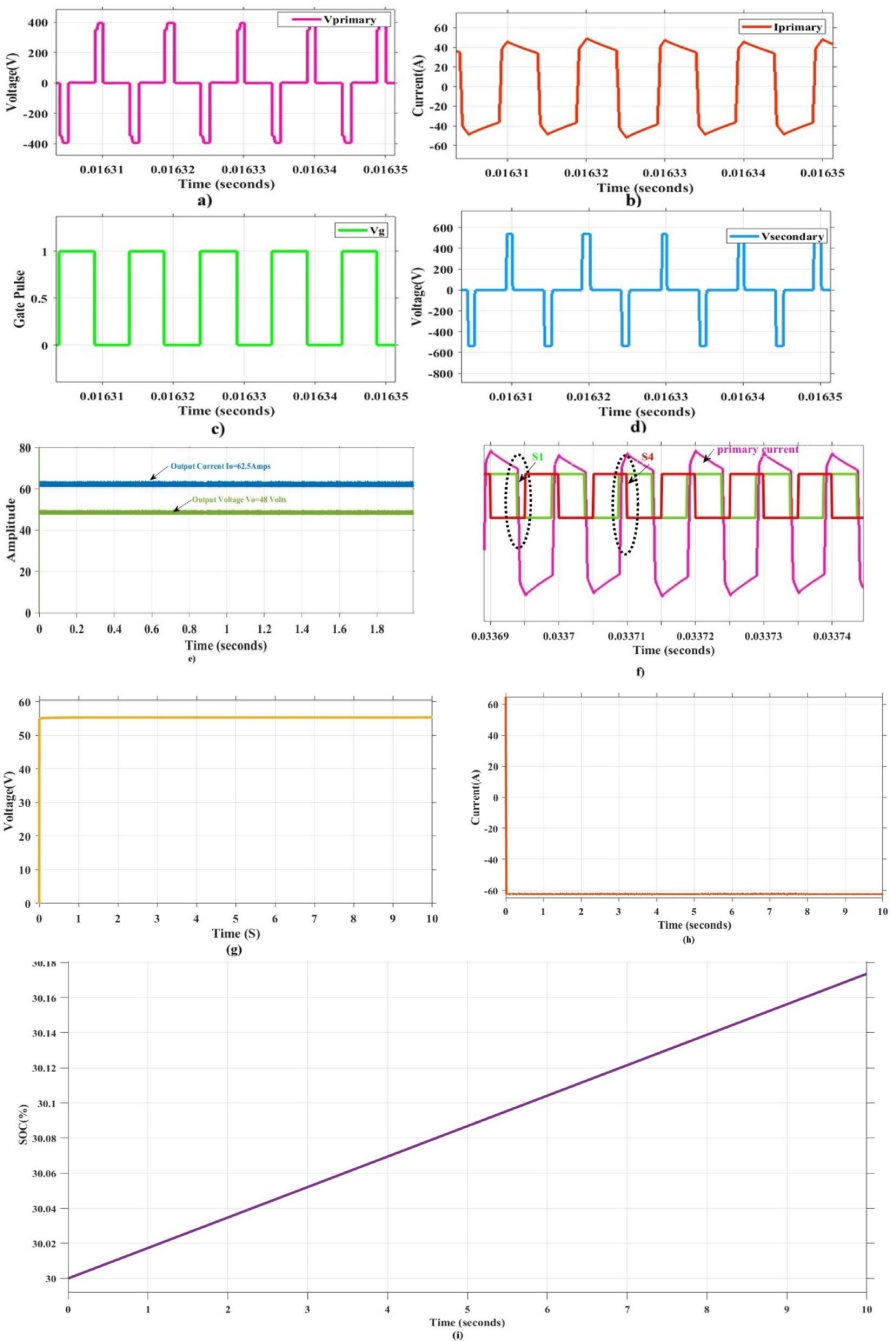
Simulation results of PSFB (**a**) Primary side voltage of High frequency transformer (**b**) Primary side current of the PSFB (**c**) Gate signal of MOSFET (**d**) Secondary side voltage of the transformer (**e**) Output voltage and Output Current (**f**) ZVS and ZCS implementation (**g**) CV mode of the battery (**h**) CC mode of the battery (**i**) 30% SoC of the battery.

The PSFB validation was conducted in MATLAB/SIMULINK with an input voltage of 400 V. The overall simulation results with three different optimization methods deployed are shown in Fig. 7. Figure 7a presents the dynamic changes in the irradiation from uniform to partial and compares the competencies of the global search algorithms. For every 2-sec there is a variation in the irradiation pattern. Throughout the full simulation duration (0–8 s), SGO consistently delivers fast convergence, minimal overshoot, and smooth transitions during step changes in power demand. The zoom view of simulation result (0–0.5 s) further emphasizes SGO’s rapid start-up response, with stable tracking of the 3000 W power target in under 0.1 s, while GWO and PSO exhibit delayed and oscillatory behaviour. Voltage regulation remains close to the target of 400 V with SGO, showing the least deviation during transients. Current tracking is similarly stable and noise-free under SGO, ensuring reduced stress on power components. The performance comparison presented in Fig. 7b includes the P&O, PSO, GWO and SGO. The simulation results clearly demonstrate the superior performance of the Social Group Optimization as it quickly reaches the maximum power point with minimal fluctuation, while P&O takes longer and shows more oscillation. The voltage and current graphs also show that SGO stabilizes faster than the others. During 0–2 s, when the irradiation is uniform, the P&O actively participates and can track the peak power 3000 W but the major drawback is the power output is oscillatory in nature. The GWO and PSO perform better than P&O but are not as fast or stable as SGO. The duty cycle graph confirms that SGO adjusts more smoothly and quickly. Overall, SGO gives the best performance with fast response and stable output, while P&O performs the worst due to slow response and high fluctuations. The Table 3 compares the performance of SGO, GWO, PSO, and P&O algorithms under uniform and partial shading conditions. It is inferred that the conventional P&O will have least power tracked and for simpler understanding if the search is related with the multi peak pattern represented in Fig. 3c, the tracked power will be only 550 W as stated in Table 4. Table 5 illustrates a comparative analysis of PSFB converter efficiency under partial shading conditions using different MPPT algorithms. The SGO algorithm achieved the highest maximum PV power of 1393 W and a corresponding PSFB output of 1261 W, resulting in the highest observed efficiency of 90.6%. GWO, PSO, and P&O also maintained similar efficiencies around 90.5%, though they extracted slightly less power from the PV source Fig. 8 illustrates the MATLAB/Simulink results of the PSFB converter. In the MATLAB simulation, ideal components such as the MOSFET, diode, high-frequency transformer, and controller are used, resulting in lossless operation. The suitable parasitic capacitance and shim inductance are chosen as 100 pF and 16 µH, respectively, for operating a 3-kW battery charging station. To ensure proper functioning of the primary and secondary voltages and currents of the high-frequency transformer, the leakage inductance of the transformer is considered as the resonant inductor. Figure 8a and b illustrate the primary-side voltage and current of the transformer. The single MOSFET gate pulse is shown in Fig. 8c. In this PSFB, ZVS is attained by utilizing the energy stored in the power transformer’s leakage inductance to softly switch each of the four power MOSFETs. Figure 8d illustrates the secondary-side current of the transformer. The simulation is verified with both a resistive load as well as battery. Figure 8e shows the output voltage and current for the resistive load. Figure 8f illustrates the achievement of ZVS in the PSFB converter with respect to S_1_ and S_4_. When the SoC is 30%, the battery charger operates in constant current (CC) mode, during which the battery voltage increases gradually. Once the battery voltage reaches 54.6 V, the converter transitions from constant current (CC) to constant voltage (CV) mode, as shown in Fig. 8g–i.

### Experimental validation results and discussions

**Fig. 9:**
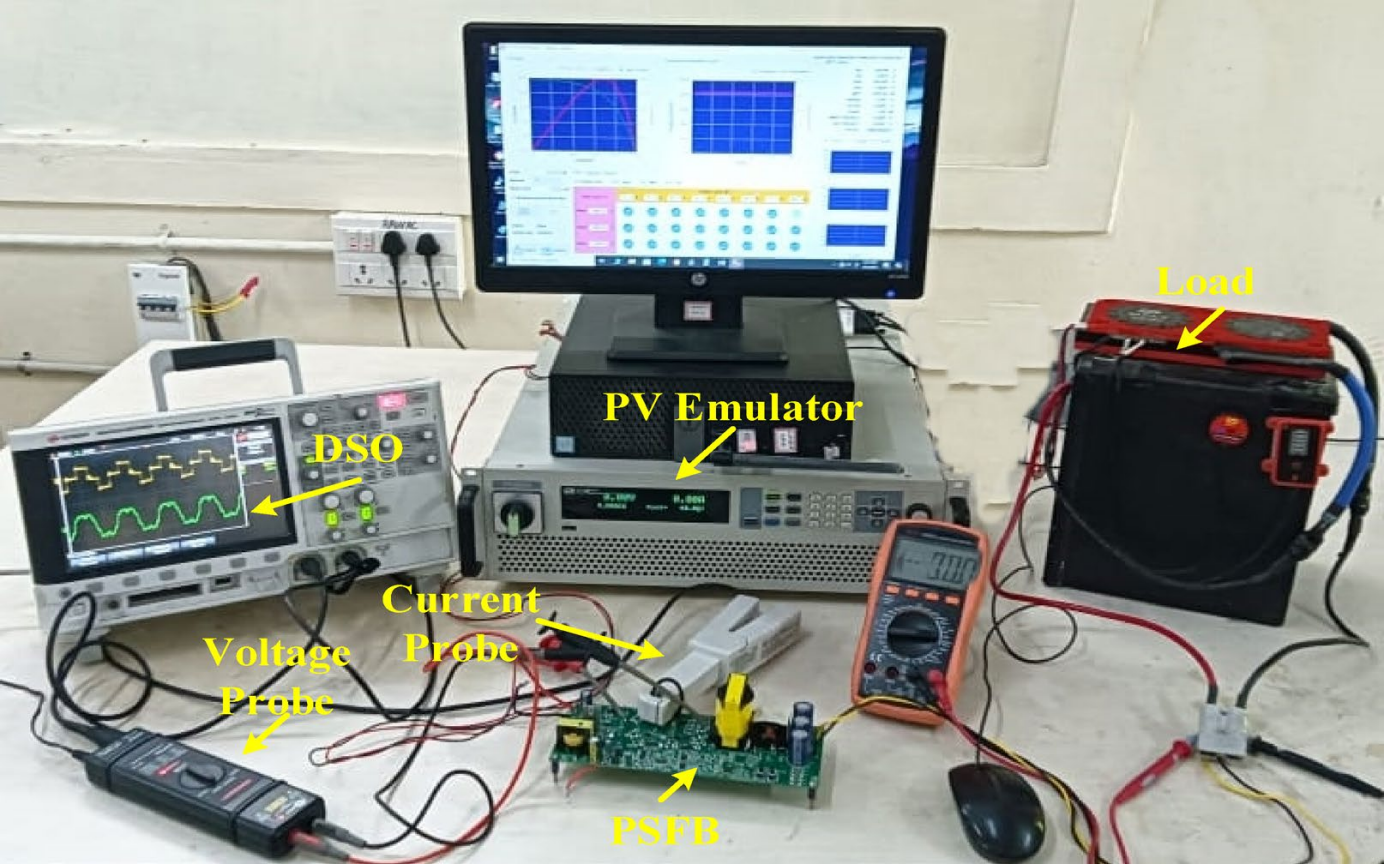
Hardware set up for measurement.

**Fig. 10:**
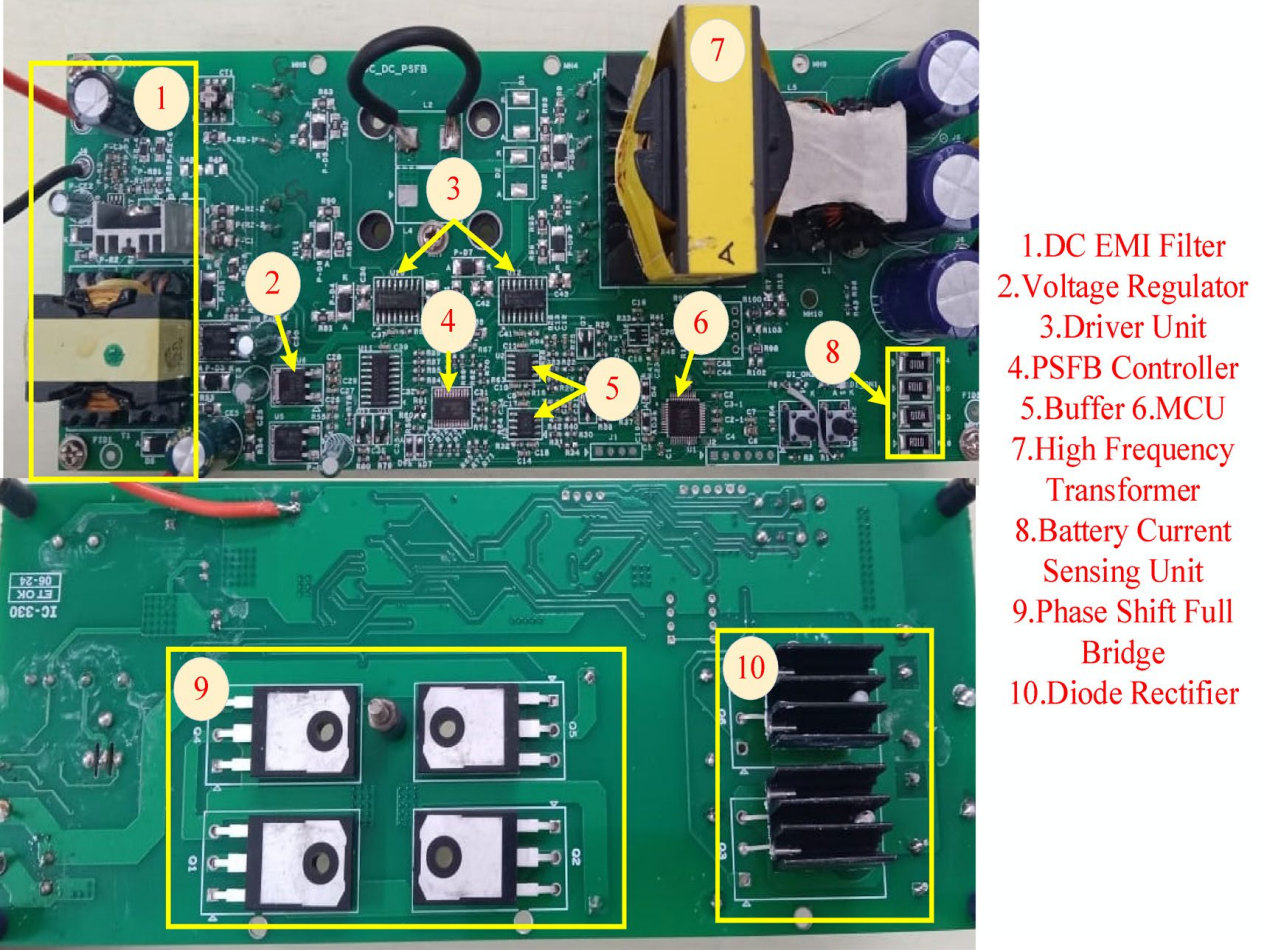
PCB layout of PSFB.

Table 2 presents design parameter of the system. The PSFB converter operates at a frequency of 100 kHz, with maximum duty cycle of 50%. Figure 9. Presents the experimental set-up of the proposed system comprising the PV emulator, PSFB converter and battery storage system. The measuring devices current probe, differential voltage probe is also presented in the Figure. The Fig. 10. illustrates a detailed schematic of a PSFB on a printed circuit board (PCB), with key components labelled for identification. The system starts with a DC EMI filter (1), which prevents electromagnetic interference from affecting the circuit. Additionally, Voltage Regulator (2), ensuring stable voltage levels for the system’s operations. The driver unit (3) controls the power transistors, enabling efficient switching, while the PSFB controller (4) generating PWM pulses to the PSFB converter. A buffer (5) has been added to stabilize the transfer of signals components. The microcontroller unit (MCU) (6) is the core processor, coordinating the overall control of the system. Energy is transferred by using the high-frequency transformer (7), isolating different sections of the circuit and adjusting voltage levels. The battery current sensing unit (8) monitors current flow to ensure efficient charging or discharging of the battery. The PSFB (9) handles high-efficiency DC-DC conversion, and finally, the diode rectifier (10) converts AC into DC to charge a battery. The emulator-based validation demonstrates the real-time feasibility of the proposed SGO-based MPPT with PSFB charging, confirming that the algorithm can be executed efficiently on embedded hardware, adapt rapidly to dynamic irradiance changes, and maintain stable converter operation with minimal oscillations, thereby improving overall energy harvesting. These outcomes suggest strong potential for deployment in practical PV-powered charging systems and scalability to larger standalone or grid-integrated applications. While the present work has been carried out using a PV emulator rather than an outdoor array, and the performance depends on appropriate tuning of algorithmic factors, these aspects mainly indicate directions for extended field validation and refinement rather than fundamental drawbacks.

**Fig. 11:**
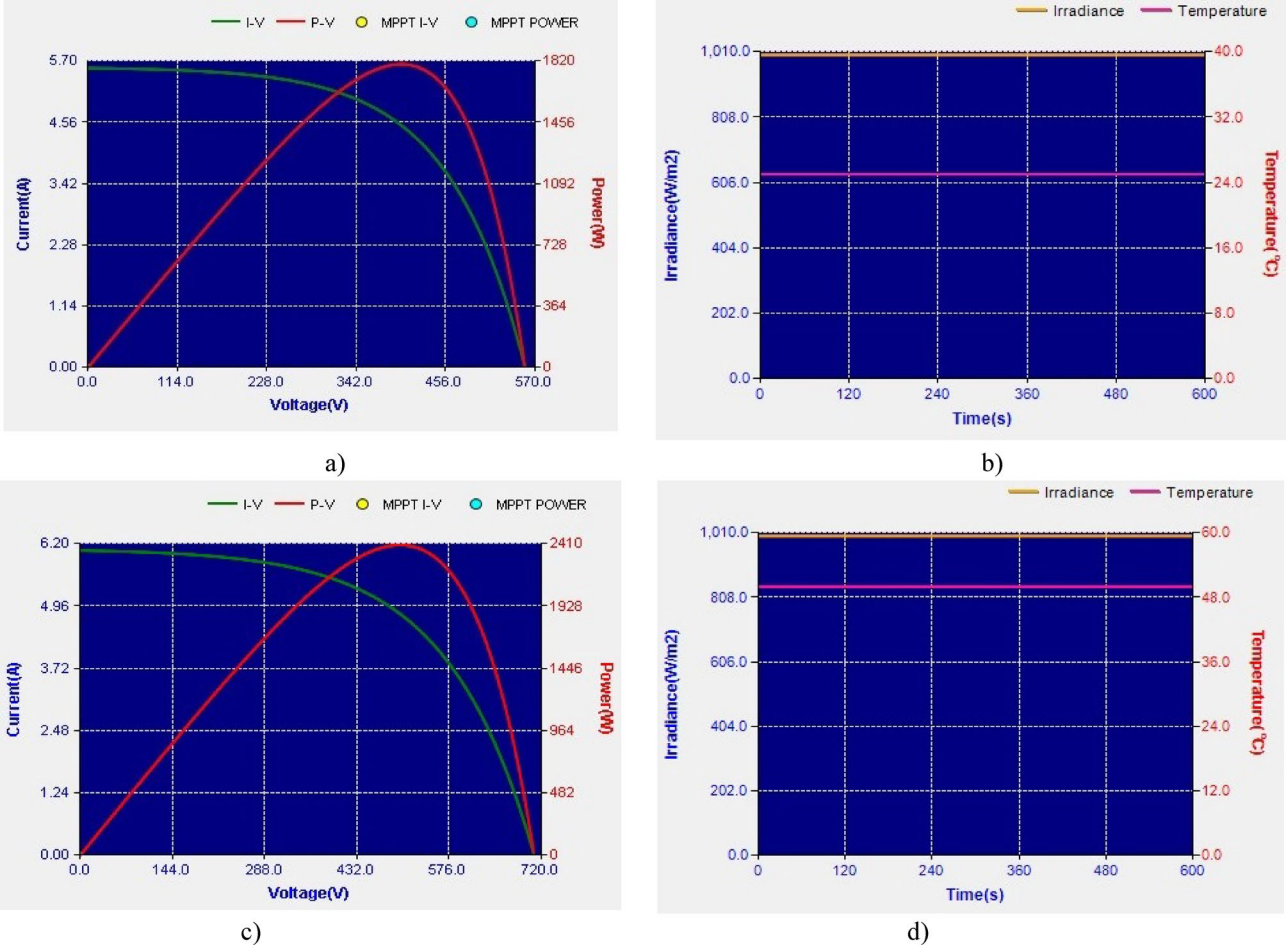
Performance characteristics of photovoltaic system (**a**) I–V and P–V curve for V_mp_=400 V (**b**) Irradiance curve at 1000 W/m^2^ (**c**) -V and P–V curve for V_mp_=500 V (**d**) Irradiance curve at 1000 W/m^2^.

The Fig. 11. presents the performance characteristics of a PV system under varying irradiance and temperature conditions. The Fig. 11a. Shows the I–V and P–V curves of the PV module. The current decreases as voltage increases, while the power initially rises, peaking at the maximum power point (MPP) before declining. The maximum current is about 5.7 A, with power peaking around 1820 W at a voltage of 430 V. The Fig. 11b. Shows constant irradiance at 1000 W/m² and temperature at 25 °C over time, indicating standard test conditions. The Fig. 11c. Shows similar I–V and P–V characteristics but at higher irradiance or temperature, with the current reaching 6.2 A and power peaking at 2400 W at a higher voltage range (500–600 V). Figure 11d. illustrates the solar irradiance remaining at 1000 W/m², while the temperature has increased to 50 °C, which provides impact the system efficiency, leading to a shift in the maximum power point.

**Fig. 12:**
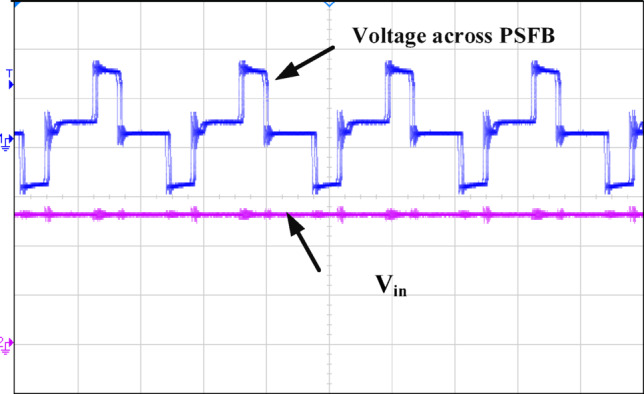
Ch1 = voltage across PSFB 200 V/divand Ch2 = output voltage of 20 V/div witht = 5 μs/div.

**Fig. 13:**
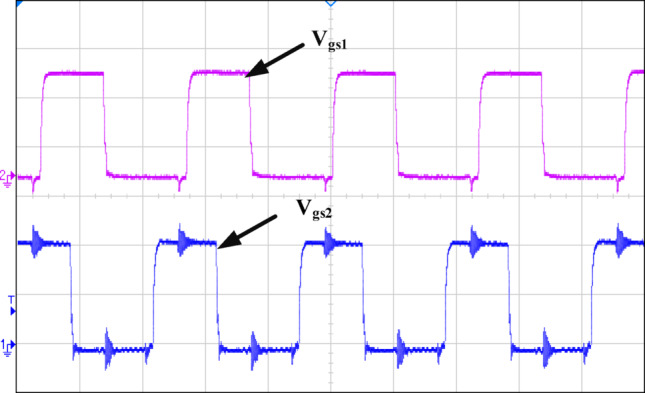
Ch1 = V_gs2_ of 200 V/div Ch2 = V_gs1_ of200 V/div.

**Fig. 14:**
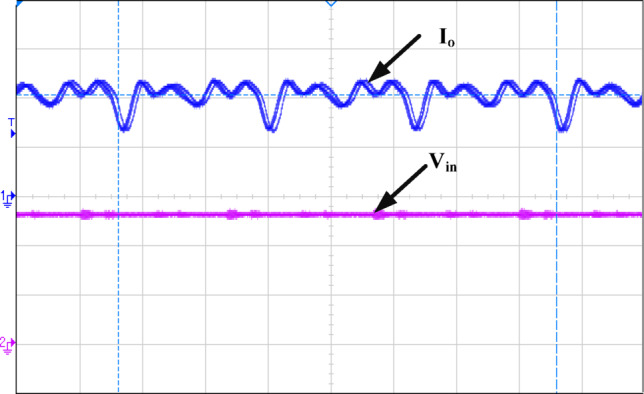
Ch1= I_o_ of 30 A/div and Ch2= V_in_ of100V/div.

**Fig. 15:**
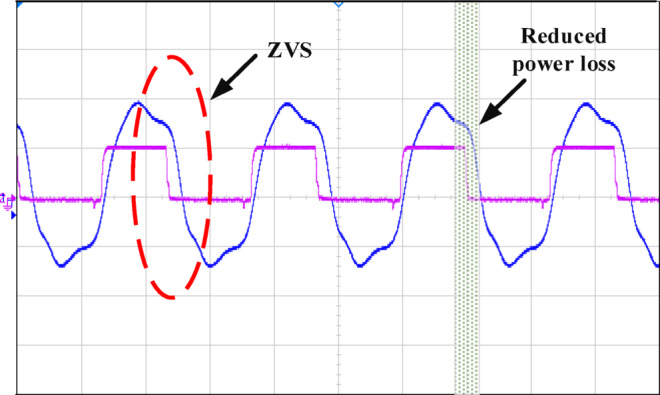
Ch1 = Ipirmary and Ch2 = gate sourcevoltage (V_gs1_).

**Fig. 16:**
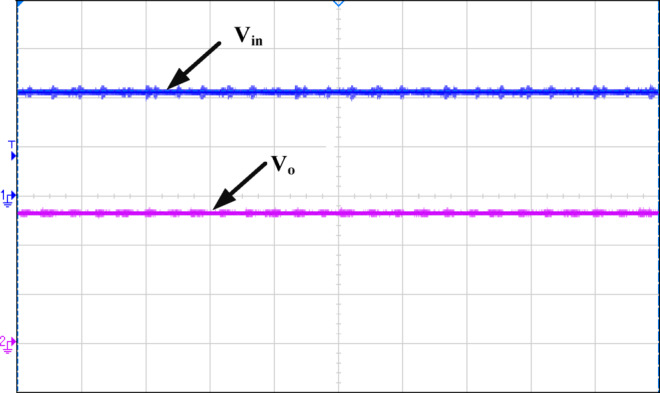
Ch1 = V_in_ of 100 V/div and Ch2 = V_o_ of20 V/div.

**Fig. 17:**
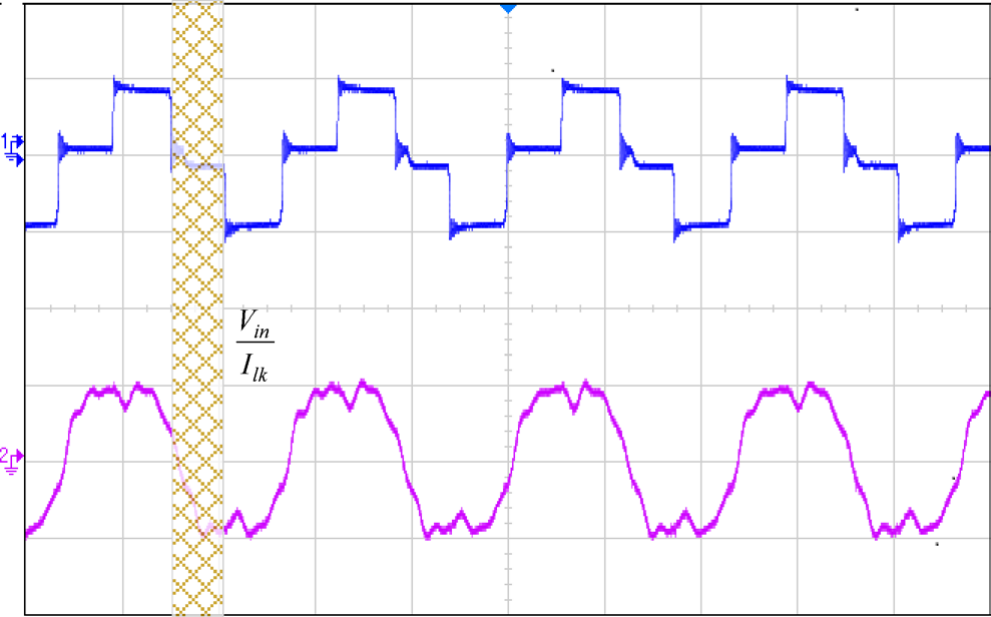
Ch1 = V_in_ of 200 V/div and Ch2= IPrimaryof 3 A/div.

**Fig. 18:**
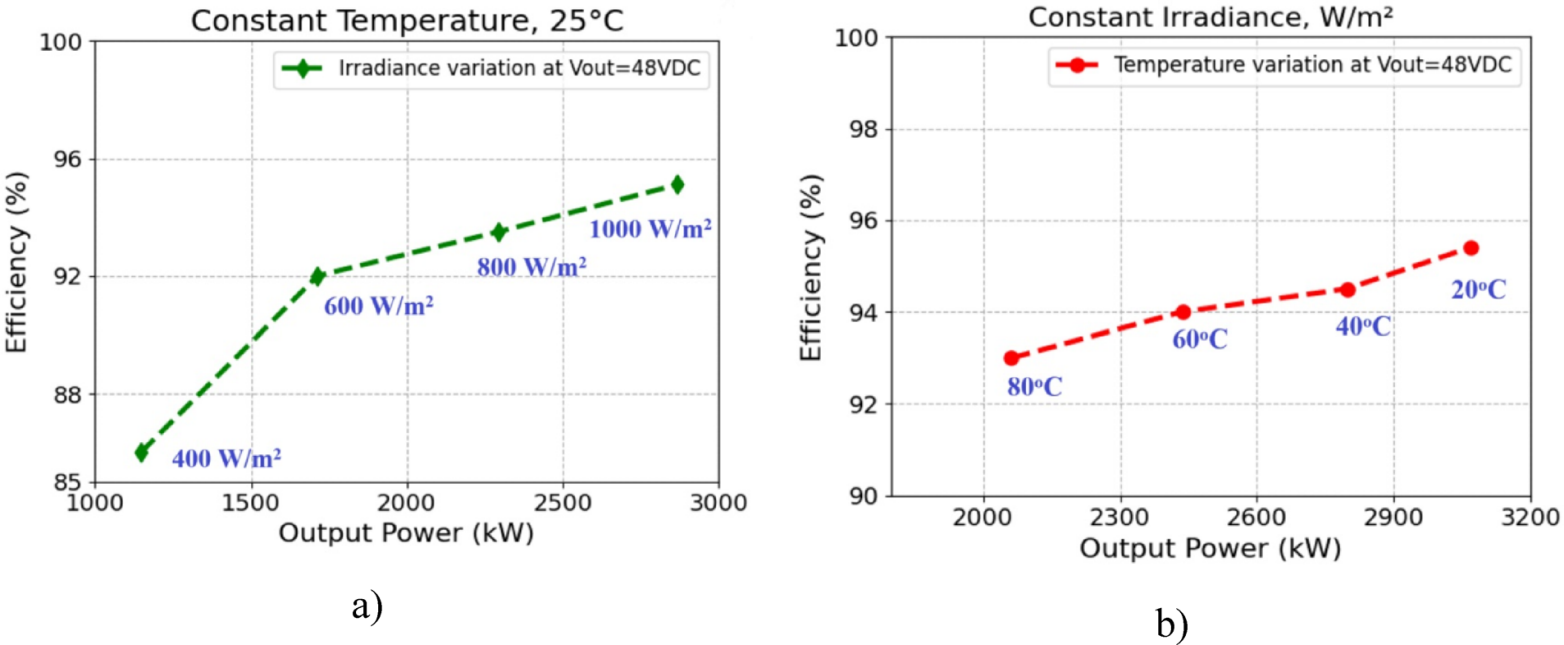
Efficiency curves of the PSFB converter for different solar panel parameters (**a**) The output power vs. efficiency of the PSFB converter for constant temperature with different irradiation (**b**) The output power vs. efficiency of the PSFB converter for constant irradiation with different temperature.

Figure 12 illustrates how the phase shift between the primary side switching signals controls the transfer of energy from the 400 V input to the transformer’s secondary side, operating with a 50% duty cycle. Figure 13 illustrates the phase-shifted gating signals of S1 and S2. Figure 14. shows that with a constant input voltage of 366 V, the output current of 62.5 A increases as the load demand rises, corresponding to a time interval of 5µs.The phase shift adjusts accordingly, regulating the amount of energy transferred to the secondary side to meet the increased load. As shown in Fig. 15. the primary side current and gate signal are depicted. The results indicate the achievement of both ZVS and ZCS. It is observed that when the primary side current is zero, the gate signal is deactivated, allowing the switch to achieve soft switching under zero current conditions. Additionally, at full load, the primary side current is higher, making it easier to achieve ZVS for the leading leg. Figure 16 shows that the varying input voltage with constant output voltage. Figure 17 illustrates the relationship between the transformer’s primary voltage and primary current. The circulating current is sustained by the transformer’s leakage and magnetizing inductance, which maintain the current flow during the freewheeling interval, even when the primary voltage is zero. Figure 18a. shows the system output power under various levels of irradiation while maintaining a constant panel temperature, along with the PSFB converter efficiency at 97%. As a result, the power increases with the irradiance, while the converter maintains an efficiency above 80%. At full load, the converter achieves 97% efficiency with reduced losses. Figure 18b. shows the system output power versus efficiency under constant irradiance and varying temperatures, with the converter maintaining an efficiency above 80%.

**Fig. 19:**
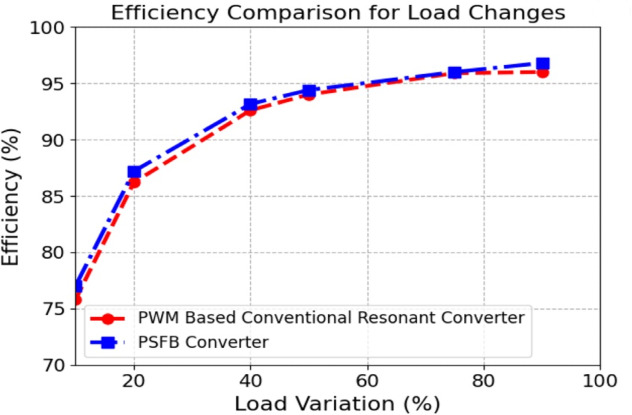
Efficiency with load variation.

Figure 19 shows the efficiency of the PSFB converter compared to the PWM-based conventional resonant converter under various load conditions. It is observed that the efficiency improves by 1% at full load and by 2% at light load due to the reduction in switching losses achieved through phase-shift control.

## Conclusion

This research work advocates a phase shift modulation and social group power tracking algorithm controlled full bridge DC-DC converter for EV application. The developed controller is highly dynamic in responding to irradiation changes and partial shading among the panels in the array. Also, the phase shift full bridge resonant converter achieves ZVS ensuring minimized losses and voltage regulation. The experimental and simulation results demonstrate that the system achieves a high efficiency of 97% under variable input voltages and maintains voltage regulation within ± 2%, ensuring stable power delivery to EV loads. These outcomes validate the effectiveness of combining intelligent control algorithms with soft-switching power converter topologies to enhance the reliability and performance of EV charging systems. However, the proposed system possess some limitations and they are : The proposed system has been developed under controlled operating conditions within tested cases. However, the uneven shading conditions may still demand fine tuning to ensure complete real-time adaptability. Also, during light loads, the ZVS margin may experience a dip which results in increased switching losses. Future scope:

4. Future work can explore integrating adaptive control techniques with the social group optimization power tracking algorithm to further enhance real-time adaptability under highly dynamic environmental conditions such as non-uniform irradiance.
5. The proposed work can be further extended by integrating multiple renewable sources through a multiport converter topology, enabling coordinated energy management across diverse inputs such as solar, wind, and battery systems. Additionally, the SGO-based MPPT algorithm can be evolved into a predictive or adaptive control framework by leveraging machine learning techniques or model predictive control (MPC) strategies.

## Data Availability

Data Availability: The datasets used and/or analysed during the current study available from the corresponding author on reasonable request.

## List of symbols

A: Random number
C: Random number
C_jd_: Junction capacitance
C_o_: Output capacitance
C_oss_: output capacitance between drain and source
C_p_: Equivalent parallel capacitance
Cr: Resonant capacitance
C1_max_, C2_max_: Cognitive parameter
C1_min_, C2_min_: Social parameter
D_MAX_: Maximum duty cycle
Fr: Resonant switching frequency
F_sw_: Switching frequency
Id: Diode current
I_Dk_: Diode current
I_mag_: Magnetising current
I_mp_: Maximum current
I_o_: Output inductor
I_pp_: Primary peak current
IPV: Saturation current
I_rms__p: Primary RMS current
I_rms__s: Secondary RMS current
k: Boltzman constant (1.38 × 10–23 J/K)
L_lk_: Leakage inductance
Lr: Resonant inductance
L_mag_: Magnetising inductance
L_o_: Output inductance
L_s_: Shim inductance
M_PV_: Maximum power of the PV panel
N_p_: Primary turns
N_s_: Secondary turns
n: Transformer turns ratio
P_o_: Output power
q: Charge of electron (1.602 × 10–19 C)
R_s_: Series resistor
R_sh_: Shunt resistor
T_HU_: Time taken to L_out_ changes from
T: Absolute temperature of the panel
V_o_: Output voltage
Vpv: Photovoltaic voltage
V_RDS_ON_: Drain source resistance
W_max_: Maximum inertia weight
W_min_: Minimum inertia weight
α: Diode ideal constant
∆I_l_OUT_: Output ripple current 90% to full load
